# JPI-547, a novel dual inhibitor of PARP1/2 and tankyrase is more effective than first-generation PARP inhibitors in preclinical BRCA1/2-mutated cancer models

**DOI:** 10.1038/s41416-026-03411-3

**Published:** 2026-05-06

**Authors:** Min Sil Kang, Nar Bahadur Katuwal, Mithun Ghosh, Sa Deok Hong, Yeong Gyu Jeong, Seong Min Park, Tae Hoen Kim, Seul-Gi Kim, Seung Ryeol Lee, Yong Wha Moon

**Affiliations:** 1 Department of Biomedical Science, The Graduate School, CHA University, Seongnam-si, Republic of Korea; 2 Department of Pathology, CHA Bundang Medical Center, CHA University, Seongnam-si, Republic of Korea; 3 Hematology and Oncology, Department of Internal Medicine, CHA Bundang Medical Center, CHA University, Seongnam-si, Republic of Korea; 4 Department of Urology, CHA Bundang Medical Center, CHA University, Seongnam-si, Republic of Korea

**Keywords:** Ovarian cancer, Breast cancer

## Abstract

**Background:**

Poly(ADP-ribose) polymerase (PARP) inhibitors are highly effective therapies for BRCA1/2-mutated tumors. However, most patients eventually develop acquired resistance. Here, we report that JPI-547, a second-generation PARP inhibitor targeting both PARP1/2 and tankyrase, demonstrates potent antitumor activity in olaparib-sensitive and resistant BRCA1/2 mutant models.

**Methods:**

Olaparib-resistant (OR) models were generated using BRCA-mutated human ovarian and breast cancer cell lines and ovarian Patient-Derived Tumor Xenograft (PDTX) by exposing to olaparib. For clinical relevance, public mRNA microarray datasets of ovarian and breast cancer were analyzed.

**Results:**

JPI-547 demonstrated better antitumor efficacy in both olaparib-sensitive and resistant BRCA-mutated preclinical models than first-generation PARP inhibitors. Mechanistically, the on-target inhibition of PARP1/2 and tankyrase by JPI-547 strongly inhibited the restoration of homologous recombination (HR) activity by suppressing RAD51 expression. This action resulted in the retardation of tumor growth in olaparib-sensitive and resistant ovarian PDTX models. Furthermore, high RAD51 expression was significantly associated with poor prognosis in ovarian and breast cancer patients based on public mRNA expression data.

**Conclusion:**

These results suggest the scientific rationale for further clinical development of JPI-547 for treating both PARP inhibitor-sensitive patients and those resistant to first-generation PARP inhibitors in BRCA-mutated cancers.

## Introduction

BRCA 1/2, known as tumor suppressors, play essential roles in HR repair [1, 2]. HR deficiency (HRD) impairs DNA damage response, which exacerbates genomic instability. Herein, PARP inhibitors can suppress BRCA1/2-mutated cancer cells effectively based on key functions of the PARP family to regulate DNA damage response [3, 4]. Considering the prevalence of BRCA1/2 mutations in various cancers, PARP inhibitors could potentially be useful in treating many types of cancer. Notably, PARP inhibitors have been studied in ovarian and breast cancers, where BRCA1/2 mutations are the most frequently observed, with an incidence of 21% in ovarian cancer and 5% in breast cancer [5].

Several PARP inhibitors, including olaparib, niraparib, rucaparib, and talazoparib, have been approved for the treatment of ovarian and breast cancer with BRCA1/2 mutation or HRD by United States Food and Drug Administration (FDA) [6]. Notably, olaparib and niraparib maintenance therapy after first-line paclitaxel/carboplatin significantly improved PFS for patients with BRCA1/2-mutated or HRD ovarian cancer in SOLO1 [7] or PRIMA [8] trials. In OLYMPIAD [9] and EMBRACA [10] trials, olaparib and talazoparib, respectively, significantly prolonged PFS in metastatic breast cancer with BRCA1/2 mutation.

Although PARP inhibitors are effective therapies for BRCA1/2-mutated cancers, acquired resistance is inevitable in most patients [5, 11]. The mechanisms of PARP inhibitor resistance include restoration of HR, stabilization of stalled replication forks, increased drug efflux, and reduction of PARP1 trapping [12, 13]. At present, no effective therapies for PARP inhibitor-resistant cancers are clinically available, although some drugs overcame resistance in preclinical studies [14, 15].

Therefore, we investigated the mechanisms underlying PARP inhibitor resistance, which could potentially be targeted for clinical drug development. Extensive research has focused on preventing HR restoration, given the critical role of BRCA1/2 in HR repair and the synthetic lethal interaction between BRCA deficiency and PARP1 inhibition. Besides PARP1, the tankyrase-MERIT40 complex is known to positively regulate HR repair by activating RAD51 [16–18]. Thus, the tankyrase-MERIT40-RAD51 pathway could be a potential target to enhance sensitivity to PARP inhibitors. JPI-547, a novel dual oral inhibitor of PARP1/2 and tankyrase, has been developed to leverage this therapeutic vulnerability [19]. JPI-547 exhibited antitumor activity in HR-deficient xenograft models [20]. Moreover, in a recent phase 1 trial, JPI-547 showed an acceptable safety profile and preliminary clinical efficacy in patients with BRCA/HRD-positive solid tumors. Of note, one of five patients (2 ovarian and 3 breast), who had ovarian cancer and stopped olaparib due to resistance, displayed a 37% reduction in tumor size [19].

Given the lack of approved therapies to restore sensitivity in the context of prevalent resistance to PARP inhibitors, we established preclinical models using BRCA1/2-mutated ovarian and breast cancer cell lines and PDTXs to study JPI-547. As a result, JPI-547 demonstrated better antitumor activity than first-generation PARP inhibitors in olaparib-sensitive and resistant preclinical models. These results provide scientific rationale for the clinical development of JPI-547 as a potential treatment for patients with BRCA 1/2-mutated cancers.

## Methods

### Drugs

JPI-547 was provided by Onconic Therapeutics Inc (South Korea). First-generation PARP inhibitors, including olaparib (cat# CS-0075), niraparib (cat# CS-0780), and talazoparib (cat# CS-0937), were purchased from ChemScene (USA). XAV-939 was purchased from Selleckchem (cat# S1180, USA).

### Cell culture

BRCA1/2-mutated olaparib-sensitive cancer cells (BT-474, SNU251, PEO.1, HCC1937 and SNU119) and olaparib-resistant cancer cells (BT-474-OR, SNU251-OR, and PEO.1-OR) were cultured in RPM1 1640 medium (cat# LM011-01, Welgene Inc., Korea) supplemented with 10% heat-inactivated FBS (cat# S001-01; Welgene Inc., Korea) and 1% penicillin-streptomycin solution (cat# LS202-02, Welgene Inc., Korea). Cells were cultured in a humidified incubator maintained at 37 °C and 5% CO_2_.

### Establishment of olaparib-resistant cancer cells

Olaparib-resistant cancer cells (BT-474-OR, SNU251-OR, and PEO.1-OR) were established by gradually increasing the olaparib concentration over 7–9 months, starting from the IC_50._ Fresh media with olaparib were changed every 3 days. Olaparib concentration was increased after the cells proliferated freely at the previous olaparib concentration. Olaparib-resistant BT-474-OR, SNU251-OR, and PEO.1-OR cells were maintained in media with 50 μM, 3.7 μM, and 20 μM of olaparib, respectively. Olaparib was washed out for 48 h before proceeding with the experiments.

### Cell viability assay

The utilized cancer cells were seeded in 96-well plates at a density of 1500–5000 cells per well, depending on the cell types, and allowed to stabilize overnight. Each PARP inhibitor was applied at different concentrations the next day and the cells were incubated for 72 h in the RPMI 1640 culture medium. The cell viability was then measured via Thiazolyl blue tetrazolium bromide assay (MTT assay, Sigma, USA), which was incubated for 4 h at 37 °C according to the manufacturer’s instructions. After incubation, the supernatant was discarded, and dimethyl sulfoxide (DMSO) was added to generate a purple formazan product for 30 min at room temperature (RT). The absorbance was then measured at 540 nm using a Multiscan GO microplate spectrophotometer. CompuSyn software (ComboSyn, USA) was utilized for the IC_50_ and combination index values.

### Quantitative real-time polymerase chain reaction (qRT-PCR)

To study the mRNA expression levels, total RNA was extracted using TRIzol (cat# 15596018, Life Technologies, USA) according to the manufacturer's instructions. The cDNA synthesis of extracted the total RNA was performed with Takara Prime Script 1st Strand cDNA Synthesis Kit (cat# 6110 A, Takara Bio Inc., Japan) following the manufacturer’s instructions. PCR amplification was carried out with Power-up SYBR Green Master Mix (cat# A25741, ThermoFisher Scientific, USA) using an ABI Step One Real-time PCR System (Applied Biosystems, UK). Each target gene expression was normalized to the β-actin or GAPDH housekeeping gene for analysis. A list of PCR primers is listed in Supplementary Table 1.

### PARP and tankyrase enzymatic activity assay

Inhibitory activity toward PARP1, PARP2, TNKS1 and TNKS2 was measured using the PARP assay kit from BPS Bioscience, following the manufacturer’s protocol. Briefly, each enzyme was incubated with NAD⁺, substrate and varying concentrations of test compound under the kit’s recommended conditions. After reaction termination and detection reagent addition, signal (chemiluminescent/fluorometric) was measured to determine percent inhibition.

### PARP1 trapping assay

Subcellular protein fractionation was carried out using the Subcellular Protein Fractionation Kit for Cultured Cells (Thermo Fisher Scientific, 78840) following the manufacturer’s protocol. Prior to fractionation, cells were treated with each PARP inhibitor at the IC_50_ concentration of JPI-547 for 4 h to assess the subcellular distribution of target proteins. Cytoplasmic and nuclear fractions were sequentially isolated and subsequently subjected to immunoblot analysis.

### Western blotting

Proteins were extracted from cells and tissues with RIPA Lysis Buffer (cat# 89901, ThermoFisher Scientific, USA) combined with a protease inhibitor cocktail (cat# 11873580001, Roche, Germany) and phosphatase inhibitors (cat# 1862495, ThermoFisher Scientific, USA). Protein concentrations were determined using the Pierce™ BCA protein assay Kit (cat# 23228 and cat# 1859078, ThermoFisher Scientific, USA). Western blotting was performed as described previously [21]. The antibodies used for western blotting are listed in Supplementary Table 2.

### Transfection with siRNA

Cells were seeded in 60 mm cell culture plates. On the following day, the cells were washed with PBS, and fresh Opti-MEM media was added. Cells were transfected with scrambled siRNA, tankyrase 1 siRNA (cat# 4390824, ThermoFisher Scientific, USA), or PARP1 siRNA (cat# 4427038, ThermoFisher Scientific, USA) using Lipofectamine RNAiMAX reagent (cat# 13778100, ThermoFisher Scientific, USA), according to the manufacturer’s instructions. The cells were incubated for 24–72 h before analyses.

### Co-immunoprecipitation (Co-IP) analysis

Samples were lysed with ice-cold NETN lysis buffer (pH 8) consisting of 250 mM NaCl, 5 mM Tris-HCL, 5 mM EDTA, 0.5% NP-40 (cat# IGEPAL CA-630, Sigma-Aldrich, USA), and protease and phosphatase inhibitor cocktails. Protein concentrations in the lysates were quantified using a Pierce^TM^BCA protein assay kit (cat# 23228 and cat# 1859078, ThermoFisher Scientific, USA). Protein lysates (containing equal protein amounts) were incubated with agarose A/G beads at 4 °C for 1 h to reduce nonspecific binding. The lysates were incubated with anti-MERIT40 antibody and protein A/G agarose beads at 4 °C overnight (cat# 20421, ThermoFisher Scientific, USA). Normal rabbit IgG was utilized as a negative control (cat# 3900, Cell Signaling Technology, USA). Immunoprecipitated proteins were analyzed using western blotting. The Co-IP antibodies are listed in Supplementary Table 2.

### Genomics of drug sensitivity (GDSC) in the cancer database analysis

Gene expression and drug screening data for the cell lines were downloaded from the GDSC website [22]. Twenty BRCA-mutated breast cancer cell lines were used to analyze the association between RAD51 expression and olaparib sensitivity, which was defined as IC_50_ of <3.8 μM. The 20 BRCA-mutated breast cancer cell lines are listed in Supplementary Table 3.

### Mice

BALB/c nude mice, nonobese diabetic/severe combined immunodeficiency (NOD/SCID) mice, and NOD SCID gamma mouse (NSG) mice were purchased from JA BIO, Inc. (Suwon, Korea). Female mice older than 6 weeks were used in all experiments. Mice were maintained in a specific pathogen-free animal facility. All possible efforts were made to minimize animal suffering.

### Animal studies

The PARP inhibitor-sensitive PDTX model, CPDX-013, was developed from an ovarian cancer patient with a BRCA2 mutation who was not exposed to PARP inhibitors. The original tumor tissue was implanted into NSG mice, resulting in the growth of a xenograft referred to as F0. The F0 xenografts were serially passaged and designated as F1, F2, F3, and so on. For this experiment, F4 tissues were transplanted into NOD/SCID mice for expansion and later transplanted into BALB/C nude mice to perform tumor growth inhibition assays. The olaparib-resistant PDTX model, WJO-003-O2, was established using WJO-003, a PARP inhibitor-sensitive PDTX model generated from an ovarian cancer patient with a BRCA1 mutation that was not exposed to PARP inhibitors. After engrafting the WJO-003 tissue, the mice were treated with 100 mg/kg olaparib twice daily. Olaparib therapy initially suppressed tumor growth, but the mice subsequently developed acquired resistance, which was defined as >25% regrowth from maximal reduction after 4 months. To evaluate the antitumor effects of JPI-547, the F4 generation of WJO-003-O2 was implanted into NOD/SCID mice.

For both PDTX experiments, mice with implanted tumor tissue were randomly divided into five groups once the tumor volume reached 60–80 mm^3^, as follows: 5 mice per group in the control and JPI-547 groups, and 10 mice per group in the olaparib, niraparib, and talazoparib, including 5 mice per group that were switched to JPI-547 when current PARP inhibitors did not suppress tumor growth. Animals in the treatment groups were orally treated with 50 mg/kg olaparib, niraparib, or JPI-547, or 0.33 mg/kg talazoparib for a maximum of 60 days, once a day. Tumor size was measured daily, and tumor volumes were calculated using the following formula: tumor length × tumor width^2^ × 0.5.

### Immunohistochemistry (IHC) staining analysis

IHC staining was performed using xenograft tumor tissues to assess RAD51 expression. The Ventana Discovery ULTRA Stainer (Roche, Germany) was used for IHC, according to the manufacturer’s instructions as previously described [21]. In brief, xenograft tumor tissue sections were stained with RAD51 primary antibody at 1:800 dilution (cat# ab133534, Abcam, USA) for 1 hour. One drop of OmniMAP anti-Rb HRP antibody (cat# 760–4310, Roche, USA) was applied for 16 min. Counterstain mounting was applied. Stained samples were observed using a light microscope. ImageJ software version 1.53 was utilized to quantify RAD51 positive cell numbers in five random nonoverlapping 400x microscopic fields for each tissue section.

### Public gene expression datasets in patients with ovarian and breast cancer

One ovarian cancer (GSE17260) and two breast cancer (GSE6532 and GSE2034) independent public mRNA expression datasets were used to evaluate the association of RAD51 mRNA expression with prognosis. The following arrays were used: Agilent-014850 Whole Human Genome Microarray 4x44K G4112F (prove name version) for GSE17260, Affymetrix Human Genome U133 Plus 2.0 Array for GF3632, and Affymetrix Human Genome U133A Array for GSE2034. Series matrix files, which were normalized by the original authors, were downloaded for our analysis. RAD51 mRNA expression levels were divided into “RAD51-Low” and “RAD51-High” groups, using the median RAD51 expression as the cut-off. PFS, recurrence-free survival (RFS), and distant RFS were analyzed for the RAD51 expression level.

### Statistical analysis

All statistical analyses were performed using SPSS version 19.0 (IBM SPSS Statistics 19.0, NY, USA). For continuous variables, student’s t-tests were used to compare two independent groups. IHC data were quantified using ImageJ software. RFS was defined as the time from curative surgery to cancer recurrence or the last date when the patient was known to be free of recurrence (censoring time). RFS, distant RFS, PFS, and OS were calculated using the Kaplan-Meier method. Log-rank tests were used to compare survival between groups. Significant prognostic variables were identified using univariate analysis, and variables with a *p*-value < 0.05 were included in the multivariate analysis using the Cox proportional hazard regression model. All p-values were two-tailed, and *p*-values < 0.05 were considered significant.

## Results

### Characterization of olaparib-resistant cells

**Fig. 1:**
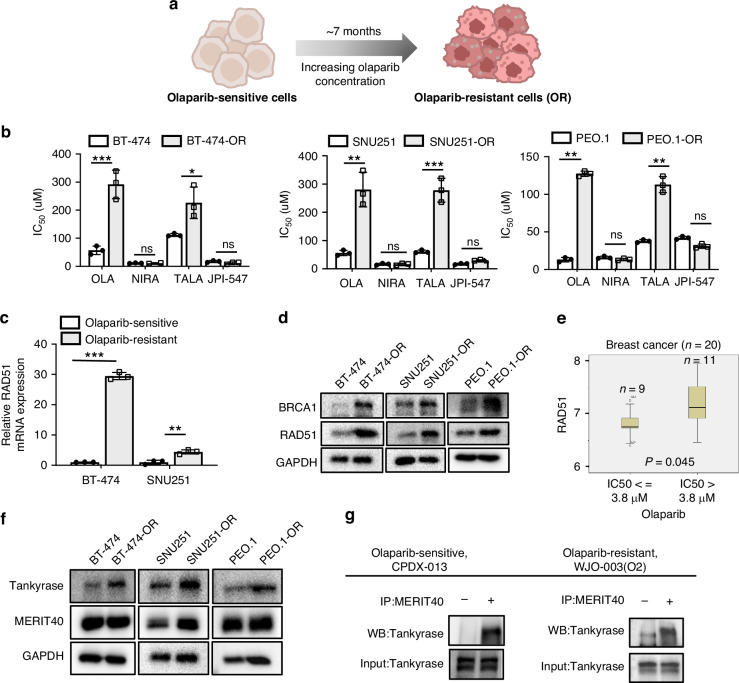
Characterization of olaparib-resistant cells. **a** schematic illustration depicting the generation of olaparib-resistant cells. **b** Comparative analysis of IC_50_ values for olaparib, niraparib, talazoparib, and JPI-547 using the MTT assay for 72 h in olaparib-sensitive cells and established olaparib-resistant cells. **c** mRNA expression of RAD51 gene in the olaparib-resistant cells compared with olaparib-sensitive cells with qRT-PCR. **d** Immunoblots showed olaparib resistance-associated protein expressions in olaparib-resistant cells compared with olaparib-sensitive cells. **e** Association between RAD51 expression and olaparib sensitivity using the GDSC database. Olaparib sensitivity was defined as IC_50_ ≤ 3.8 uM and IC_50_ > 3.8 μM in the BRCA1/2-mutated breast cancer cell lines. *p*-value was calculated by independent sample t-test. **f** Immunoblots showed tankyrase and MERIT40, comparing the olaparib-sensitive cells to their corresponding olaparib-resistant cells. **g** CO-IP assay showing the complex formation of tankyrase and MERIT40 in olaparib-sensitive model (CPDX-013 ovarian cancer PDTX) and olaparib-resistant model (WJO-003-O2 ovarian cancer PDTX).

Olaparib-resistant ovarian and breast cancer cells were generated as described in the methods section (Fig. 1a). The IC_50_ values for olaparib increased significantly in the olaparib-resistant cells (291.7 µM in BT-474-OR, 280.7 µM in SNU251-OR, and 127.5 µM in PEO.1-OR) relative to their corresponding olaparib-sensitive cells (57.2 µM in BT-474, 56.2 µM in SNU251, and 13.1 µM in PEO.1) (Fig. 1b and Supplementary Fig. 1A). The olaparib-resistant cells exhibited cross-resistance to talazoparib (Fig. 1b). Intriguingly, the IC_50_ values for niraparib or JPI-547 were relatively unchanged in olaparib-resistant cells compared with the corresponding olaparib-sensitive cells. This may be associated with their higher cellular permeability, as reflected by greater apical-to-basolateral apparent permeability coefficients (Papp) for niraparib (11.6 × 10⁻⁶ cm/s at 37.5 μM) and JPI-547 (18.1 × 10⁻⁶ cm/s at 10 μM) compared with olaparib (3.67 ± 0.34 × 10⁻⁶ cm/s at 10 μM) [23–26]. Furthermore, previous reports demonstrated that niraparib retains efficacy in olaparib-resistant BRCA1-mutant ovarian cancer cells by inducing greater replication stress, independent of drug efflux mechanisms [27]. Niraparib has also been reported to reach higher concentrations in tumor tissue, exhibit distinct PARP-trapping characteristics, and inhibit pSTAT3 compared with olaparib, which may account for its enhanced potency in this setting [24, 25]. Collectively, these findings suggest that the lack of cross-resistance to niraparib and JPI-547 may be attributed not only to their favorable pharmacological and permeability profiles, but also to the heterogeneous nature of the mechanisms driving olaparib resistance acquisition.

Recent evidence suggests that HR restoration is a significant factor in the acquired resistance to PARP inhibitors [28, 29]. Therefore, the expression of HR-associated key proteins, including BRCA1 and RAD51, was further analyzed. BRCA1 and RAD51 increased significantly in olaparib-resistant cells compared with the corresponding olaparib-sensitive cells (Fig. 1c, d). These results indicate that HR restoration plays a role in acquiring olaparib resistance. Thus, the relationship between RAD51 expression and olaparib activity in 20 BRCA1/2-mutated breast cancer cell lines from the GDSC database was analyzed (Fig. 1e). Olaparib activity significantly and inversely correlated with RAD51 expression (*P* = 0.045); RAD51 expression was higher in cells with lower olaparib activity (IC_50_ > 3.8 µM). As stated in the introduction, RAD51 plays a crucial role in the final stages of the HR, and its overexpression has been implicated in resistance to PARP inhibitors. To further investigate this mechanism, we evaluated HR-related genes that regulate RAD51 expression. Based on a previous study, tankyrase and MERIT40 are known to positively regulate HR repair [30]. Our analysis revealed that tankyrase expression was slightly elevated in olaparib-resistant cells, and MERIT40 protein levels increased in both olaparib-sensitive and resistant cells (Fig. 1f). Moreover, recent evidence suggests that tankyrase interacts with MERIT40 to stimulate HR [30, 31]. Consistent with this, CO-IP assay confirmed the complex formation between tankyrase and MERIT40 in both the olaparib-sensitive (ovarian cancer PDTX CPDX-013) and olaparib-resistant (ovarian cancer PDTX WJO-003-O2) models (Fig. 1g).

### JPI-547 is a potent and selective inhibitor for PARP1/2 and tankyrase

Based on these findings, we hypothesized that targeting tankyrase-MERIT40 pathway alongside PARP inhibition could enhance the antitumor efficacy of PARP inhibitors. To test this, we further evaluated the JPI-547, a novel dual inhibitor of PARP1/2 and tankyrase. The effectiveness of JPI-547 as a dual inhibitor of PARP1/2 and tankyrase was compared with conventional first-generation PARP inhibitors (olaparib, niraparib, and talazoparib).

**Fig. 2:**
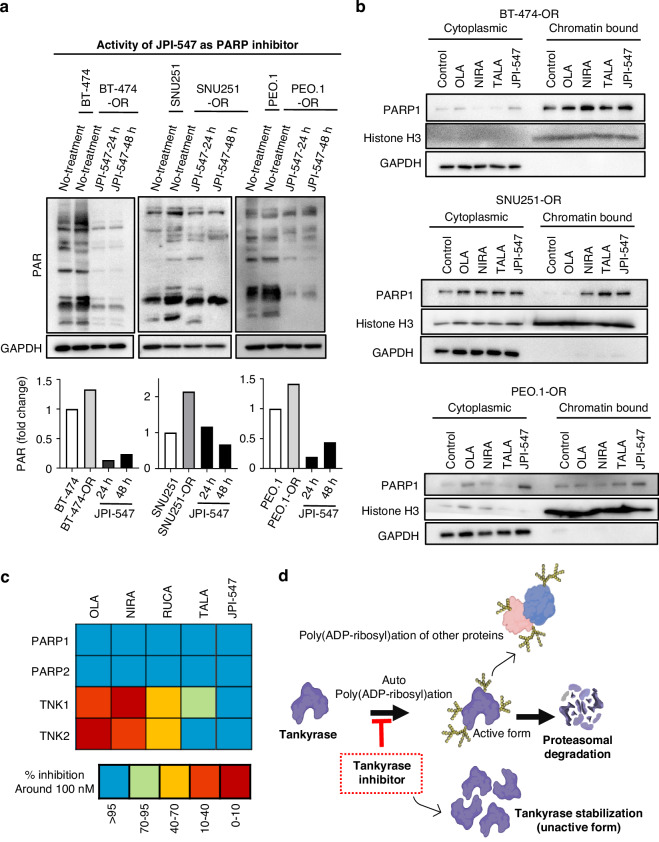

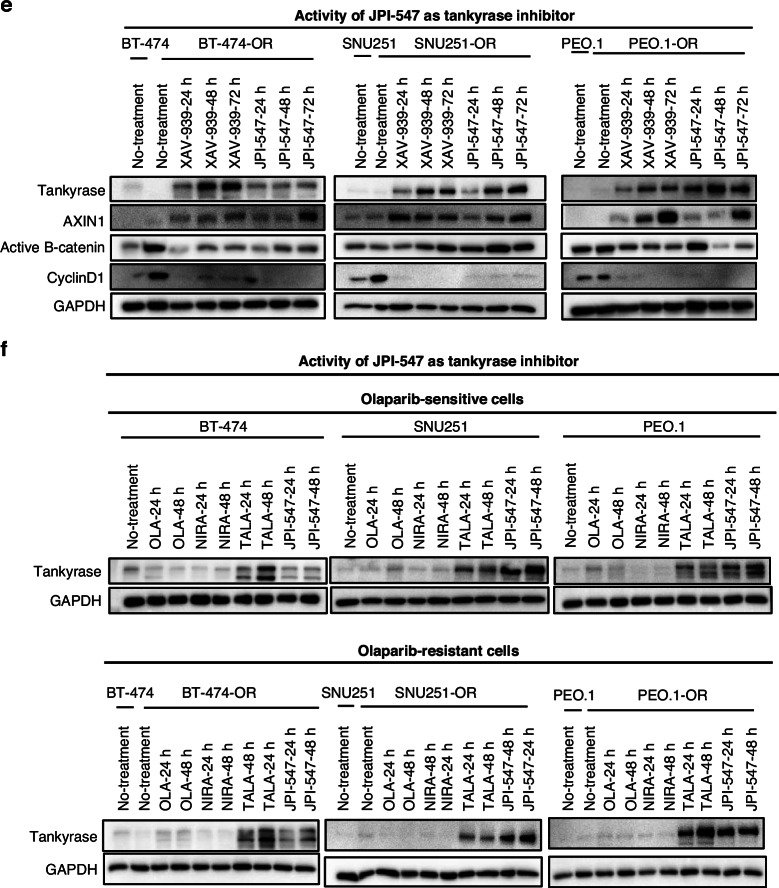
JPI-547 is a potent and selective inhibitor for PARP1/2 and tankyrase. **a** Representative immunoblots analysis showing the detection of PAR (poly(ADP-ribose)) in olaparib-resistant cells after treatment with JPI-547 for 24 and 48 h at respective IC_50_ concentrations. **b** Representative immunoblots analysis showing the detection of PARP1 trapping in olaparib-resistant cells following 4 h treatment with each PARP inhibitor at JPI-547’s IC_50_ concentration determined for each olaparib-resistant cell. **c** Heatmap visualizing enzymatic activity screening 5 PARP inhibitors against 4 PARP enzymes at 100 nM concentration. **d** A schematic illustration depicting the mechanism of tankyrase action. **e** Comparative analysis of the inhibitory activities of XAV-939 (selective tankyrase inhibitor, positive control) and JPI-547 on tankyrase auto-PARylation and WNT/β-catenin pathway–associated proteins in olaparib-resistant cells. **f** Comparative analysis of tankyrase auto-PARylation inhibition using four distinct PARP inhibitors. Three olaparib-sensitive cells and their corresponding olaparib-resistant cells were incubated with IC_50_ concentrations of each PARP inhibitor for 24 and 48 h.

To first assess the PARP-inhibitory activity of JPI-547, we examined total cellular PARylation in olaparib-resistant cells (Fig. 2a). Treatment with JPI-547 resulted in a significant reduction in PAR levels, indicating effective suppression of PARP activity (Fig. 2a). Then, we examined whether first-generation PARP inhibitors can also reduce PARylation levels in olaparib-resistant cells. As shown in Supplementary Fig. 2A, clinically approved first-generation PARP inhibitors efficiently decreased cellular PAR formation in olaparib-resistant cells to a level comparable to JPI-547. Thus, suppression of PARylation itself is not unique to JPI-547. Importantly, however, despite a similar ability to inhibit PARylation, JPI-547 exhibited a superior antitumor capacity in our preclinical olaparib-resistant models compared with first-generation PARP inhibitors. These findings suggest that other mechanisms beyond simple catalytic inhibition of PARP may contribute to the enhanced activity of JPI-547 in olaparib-resistant cells. To further evaluate PARP1 trapping, olaparib-resistant cells were treated with each PARP inhibitor at IC₅₀-matched concentrations of JPI-547 (Fig. 2b). JPI-547 treatment resulted in a consistent increase in chromatin-associated PARP1 compared with vehicle control across all olaparib-resistant models, indicating enhanced PARP1 retention on DNA. Under these conditions, the overall PARP1-trapping capacity of JPI-547 was comparable to that observed with niraparib and talazoparib, with modest cell line–dependent variability. Collectively, these results indicate that the PARP1-trapping activity of JPI-547 is comparable to that of clinically established PARP inhibitors in olaparib-resistant models.

Next, we assessed the tankyrase-inhibitory activity of JPI-547. First, we performed an enzymatic inhibition assay across PARP family members, including PARP1, PARP2, and tankyrase (Fig. 2c). The assay included four FDA-approved PARP inhibitors (olaparib, niraparib, rucaparib, and talazoparib) for comparison purposes. All reference PARP inhibitors strongly inhibited PARP1 and PARP2 (>95%) at 100 nM, while exhibiting limited activity against tankyrase. In contrast, JPI-547 selectively inhibited tankyrase. Notably, among the tested PARP inhibitors, talazoparib displayed moderate inhibition of tankyrase, although its potency was weaker than that of JPI-547. Therefore, these findings indicate that JPI-547 possesses a distinct selectivity profile, preferentially targeting tankyrase enzymes within the PARP family. Tankyrase is known to regulate protein stability, including itself, via PARylation-dependent ubiquitination [31]. Therefore, tankyrase inhibitors prevent degradation of tankyrase through the ubiquitin-proteasome pathway, resulting in the accumulation of the protein within the cell as shown in Fig. 2d. Thus, auto-PARylation inhibition capacity of tankyrase after treatment with selective tankyrase inhibitor, XAV-939 (positive control), and JPI-547 was assessed in olaparib-resistant cells (Fig. 2e). Tankyrase protein levels increased significantly in olaparib-sensitive (Supplementary Fig. 2B) and olaparib-resistant cells as well, responding to both XAV-939 and JPI-547 compared with protein levels in the no treatment control. This result indicated decreased tankyrase auto-PARylation and subsequent stabilization [32]. We next examined the effect of JPI-547 on the WNT/β-catenin signaling pathway (Fig. 2e), given prior research demonstrating that JPI-547 suppresses Wnt/β-catenin signaling through tankyrase inhibition in Wnt-addicted cancers [20]. Similar to the tankyrase inhibitor XAV-939, JPI-547 treatment led to stabilization of AXIN1 and a concomitant reduction in cyclin D1 expression. Although the change in active β-catenin was modest, the stabilization of AXIN1 alongside reduced cyclin D1 expression is consistent with a partial dampening of WNT/β-catenin pathway activity. The tankyrase inhibitory activity of the four different PARP inhibitors was further evaluated by measuring the inhibition of tankyrase auto-PARylation (Fig. 2f). Both JPI-547 and talazoparib inhibited tankyrase auto-PARylation in the olaparib-sensitive and olaparib-resistant cells [33]. These results confirmed that JPI-547 inhibits tankyrase activity.

Taken together, JPI-547 exhibited potent dual inhibition of PARP1/2 and tankyrase in ovarian or breast cancer cells harboring BRCA1/2 mutations.

### JPI-547 more effectively disrupts HR repair than first-generation PARP inhibitors

**Fig. 3:**
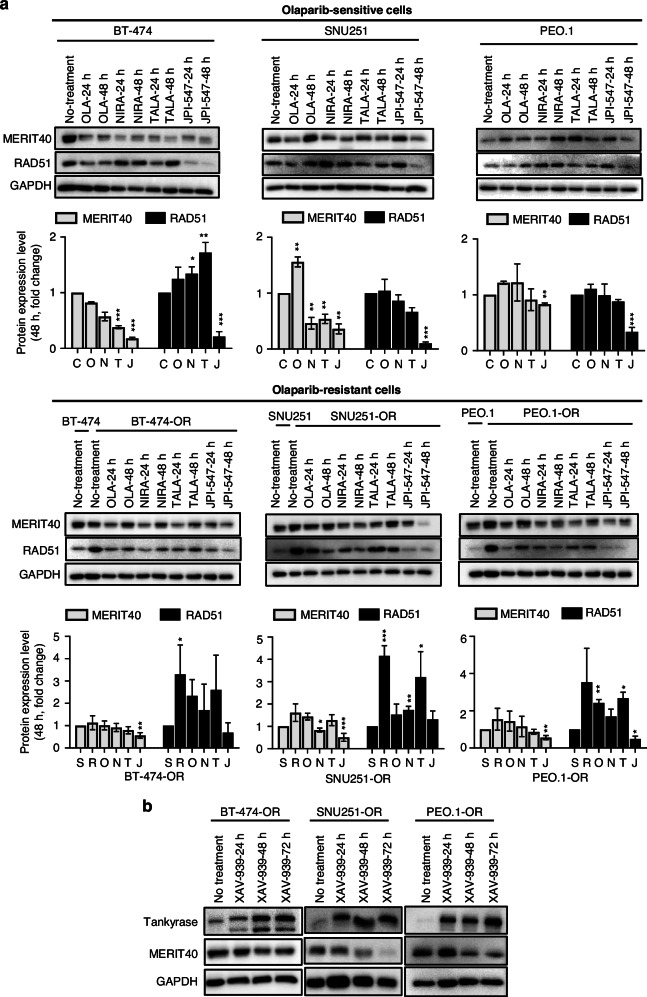

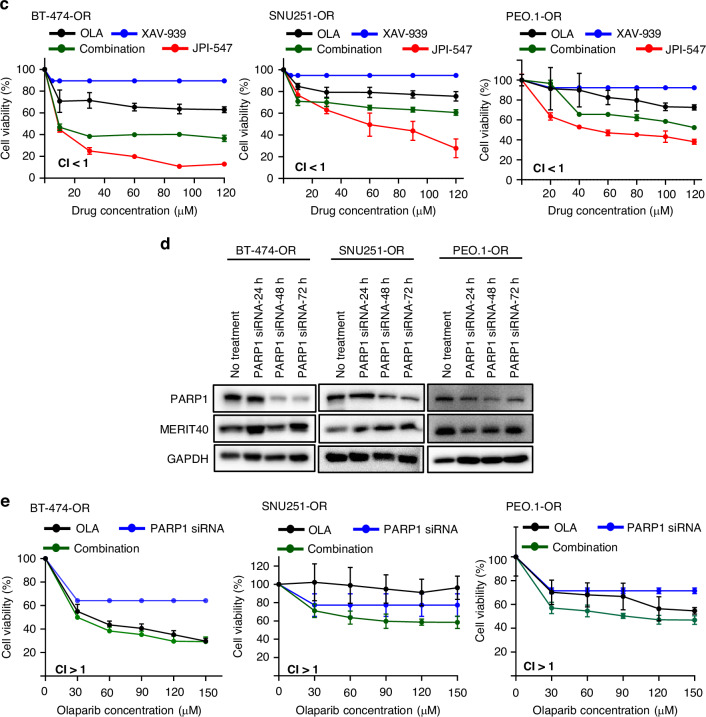
JPI-547 effectively disrupts HR repair than first-generation PARP inhibitors. **a** Immunoblot analysis of HR-associated gene expression levels. Three olaparib-sensitive cells were treated with each PARP inhibitor at its IC_50_ concentration. In contrast, their corresponding olaparib-resistant cells were treated with each PARP inhibitor at the IC_50_ concentration of JPI-547, as determined for each olaparib-resistant cell line, for 24 and 48 h (S: olaparib-sensitive, R: olaparib-resistant, O: olaparib, N: niraparib, T: talazoparib, J: JPI-547). **b** Immunoblot analysis of indicated proteins in olaparib-resistant cells after treatment with 10 uM of XAV-939 for 24–72 h. **c** Cell viability assay (MTT) of olaparib-resistant cells after treatment with XAV-939 or olaparib or JPI-547 or combination of various concentrations of olaparib and a fixed concentration of XAV-939 (5 uM) for 72 h. **d** Immunoblot analysis of indicated proteins in olaparib-resistant cells after treatment with PARP1 siRNA for 24–72 h. **e** Cell viability assay (MTT) of olaparib-resistant cells after treatment with PARP1 siRNA or olaparib and combination of various concentrations of olaparib and a fixed ratio of PARP1 siRNA for 72 h.

We further elucidated mechanisms underlying JPI-547. We investigated whether the dual inhibition of PARP1/2 and tankyrase influences the expression of HR-related genes. Specifically, we examined the expression levels of MERIT40 and RAD51 in both olaparib-sensitive and resistant cells after treatment with various PARP inhibitors. Each olaparib-sensitive cell was treated with the corresponding PARP inhibitor at its own IC_50_ concentration. In contrast, each olaparib-resistant cell was treated with each PARP inhibitor at the IC_50_ concentration of JPI-547 specific to that resistant cell. Intriguingly, JPI-547 suppressed the expression of MERIT40, tankyrase complex partner, and RAD51 proteins in both olaparib-sensitive cells and olaparib-resistant cells. Previous work demonstrated that tankyrase can recruit MERIT40 to chromatin [17]. Further mechanistic studies will be required to clarify how MERIT40 expression and stability are controlled under DNA damage or PARP inhibition conditions. In parallel, RAD51 suppression with JPI-547 was more pronounced compared to the no-treatment control or treatment with other PARP inhibitors (Fig. 3a, Supplementary Fig. 3A). Therefore, our findings indicate that dual inhibition of PARP1/2 and tankyrase with JPI-547 eventually suppresses RAD51, which plays a central role in the final step of the HR process. Although additional analyses, such as a DR-GFP reporter assay measuring HR efficiency, would be needed to further validate this observation.

Next, we investigated whether tankyrase or PARP1 regulates MERIT40 in the HR process above RAD51. We found that XAV-939, selective tankyrase inhibitor, stabilized tankyrase and subsequently decreased MERIT40 expression in olaparib-resistant cells for different periods of 24–72 h (Fig. 3b) [17]. To further explore whether inhibition of tankyrase synergizes with olaparib, we performed a proliferation assay in olaparib-resistant cells following treatment with olaparib or XAV-939, or their combination [34]. For comparison purposes, JPI-547 was also included in the treatment (Fig. 3c).

We then investigated whether PARP1 inhibition would affect the expression of MERIT40 and its synergistic effect when combined with olaparib in olaparib-resistant cells (Fig. 3d, e). Selective inhibition of PARP1 with siRNA did not suppress the MERIT40 in olaparib-resistant cells (Fig. 3d). As expected, PARP1 siRNA combined with olaparib did not show synergistic antiproliferative activity in olaparib-resistant cells, indicating further inhibition of PARP1 did not affect resensitization to olaparib (Fig. 3e). Taken together, JPI-547 enhanced resensitization by impairing HR restoration via the tankyrase-MERIT40-RAD51 axis.

Collectively, our findings suggest that JPI-547 effectively suppressed RAD51 through the modulation of MERIT40, thereby inhibiting the HR repair process.

### JPI-547 treatment leads to greater tumor regression than first-generation PARP inhibitors in the olaparib-sensitive ovarian cancer PDTX model

**Fig. 4:**
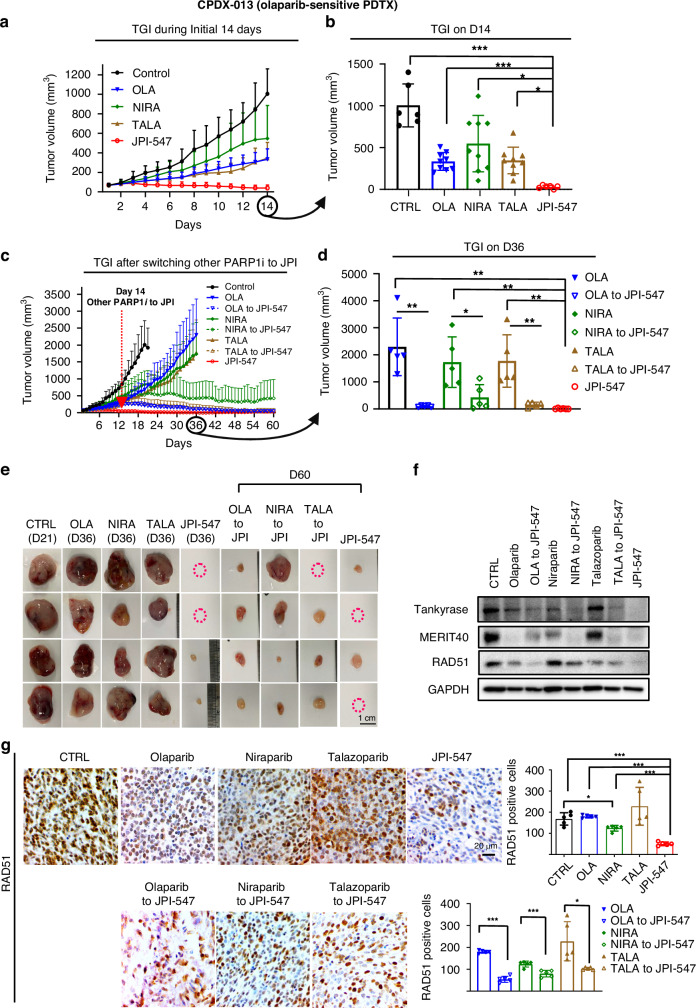
JPI-547 treatment leads to greater tumor regression than first-generation PARP inhibitors in the olaparib-sensitive ovarian cancer PDTX model. **a** Line graphs of average tumor volume in each PARP inhibitor treatment group before switching to JPI-547 (day 14). **b** Bar graphs of average tumor volume in each PARP inhibitor treatment group before switching to JPI-547 on day 14. Independent sample t-test results: *p < 0.05, **p < 0.01, ***p < 0.001. **c** Line graphs of average tumor volume in the indicated treatment groups. In 5 of the ten mice from the groups with first-generation PARP inhibitor treatment (olaparib, niraparib, talazoparib), each first-generation PARP inhibitor was switched to JPI-547 on day 14 and then JPI-547 was continuously administered upto 60 days. **d** Bar graphs of average tumor volume in each PARP inhibitor treatment group after switching to JPI-547 on day 36 (*n* = 5). **e** Images of harvested tumor tissues. The red dotted circles represent the complete regression of the tumor. **f** Immunoblot analysis of tumor tissues showing comparative expressions of tankyrase, MERIT40, and RAD51 in each group. **g** Immunohistochemistry staining of RAD51 in CPDX-013 engraft tumors of indicated groups. Staining images were collected at 400x magnification. The bar graph represented the RAD51 protein expression in 5 random, non-overlapped fields. Independent sample t-test: *p < 0.05, **p < 0.01, ***p < 0.001.

Based on our findings, we next aimed to determine whether JPI-547 could suppress tumor growth. First, the olaparib-sensitive PDTX model, CPDX-013, was established from human ovarian cancer with BRCA2 mutation. JPI-547 and first-generation PARP inhibitors were administered every day for 14 days. On day 14, JPI-547 was administered in half of the mice receiving first-generation PARP inhibitors to evaluate the antitumor activity of JPI-547. During the individual PARP inhibitor treatments, significantly greater tumor regression was observed in the JPI-547 treatment group compared with tumor growth in the first-generation PARP inhibitor groups (Fig. 4a, b). Intriguingly, after switching from first-generation PARP inhibitors to JPI-547, tumor reduction was significantly greater in the groups switched to JPI-547 than the corresponding first-generation PARP inhibitor maintenance groups (Fig. 4c, d). Importantly, none of the treatments caused weight loss, suggesting the absence of systemic drug toxicity (Supplementary Fig. 4A). Notably, in the JPI-547 treatment group, four out of 7 mice (57.1%) exhibited complete tumor regression on day 60.

Mice were sacrificed and tumors were excised after 60 days of drug treatment (Fig. 4e). Then protein expression levels were analyzed in xenograft tumor tissues (Fig. 4f and Supplementary Fig. 4B). Consistent with the in vitro findings, PAR, tankyrase, MERIT40, and RAD51 protein levels, which were measured by immunoblot were suppressed in the treatment groups switched to JPI-547 from first-generation PARP inhibitor treatments. The changes in RAD51 expression were confirmed with IHC (Fig. 4g). RAD51 expression was significantly decreased in the JPI-547 treatment group compared with expression in the control (*p* < 0.001) or other PARP inhibitor (*p* < 0.001) groups. Furthermore, switch-to-JPI-547 groups showed significant RAD51 suppression compared to non-switch groups which received only first-generation PARP inhibitors. These results further supported the in vitro results showing that JPI-547 exhibited greater antitumor activity than the other first-generation PARP inhibitors by inhibiting HR restoration in a PARP inhibitor-sensitive model.

### JPI-547 treatment leads to greater tumor regression than first-generation PARP inhibitors in the olaparib-resistant ovarian cancer PDTX model

After we confirmed that JPI-547 has the greatest antitumor effect in the PARP inhibitor-sensitive model, we further explored the antitumor potential of JPI-547 in the olaparib-resistant PDTX model (WJO-003-O2). As described in the methods section, we generated an olaparib-resistant PDTX model from a PARP inhibitor-sensitive ovarian PDTX model, WJO-003 tissue derived from an ovarian cancer patient with BRCA1 mutation. Mice were administered with first-generation PARP inhibitors every day. After 21 days of treatment, half of the mice in each PARP inhibitor group were switched to JPI-547 treatment to evaluate its anti-tumor activity in olaparib-resistant ovarian cancer, which was previously exposed to first-generation PARP inhibitors treatment.

**Fig. 5:**
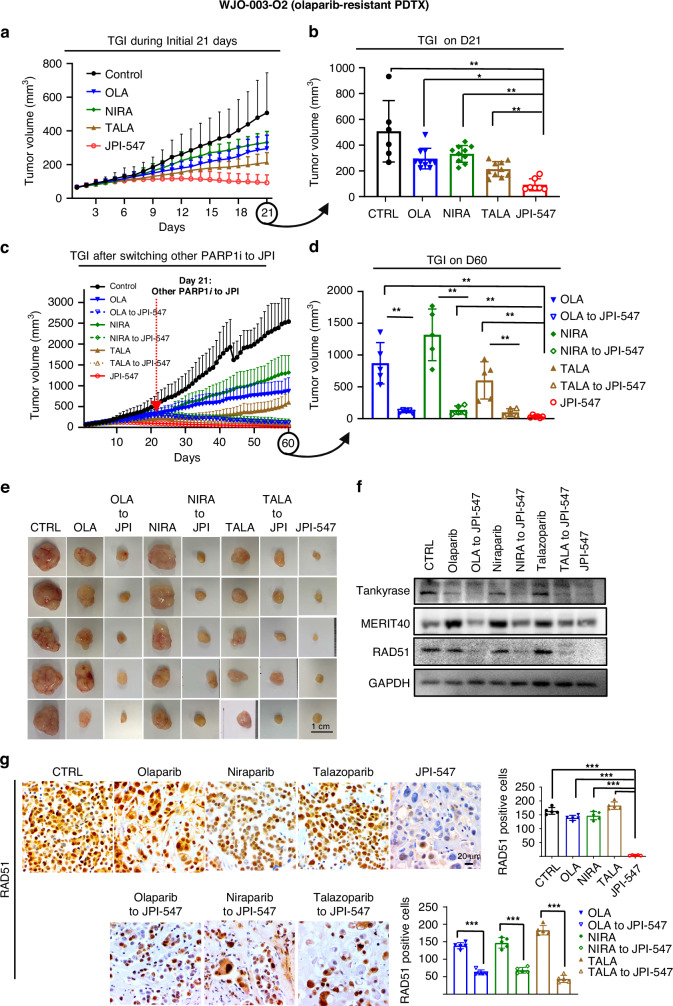
JPI-547 treatment leads to greater tumor regression than first-generation PARP inhibitors in the olaparib-resistant ovarian cancer PDTX model. **a** Line graphs of average tumor volume in each PARP inhibitor treatment group before switching to JPI-547 (day 21). **b** Bar graphs of average tumor volume in each PARP inhibitor treatment group before switching to JPI-547 on day 21. Independent sample t-test results: *p < 0.05, **p < 0.01, ***p < 0.001. **c** Line graphs of average tumor volume in the indicated treatment groups. In 5 of the ten mice from the groups with first-generation PARP inhibitors (olaparib, niraparib, talazoparib), each PARP inhibitor was switched to JPI-547 on day 21 and then JPI-547 was continuously administered upto 60 days. **d** Bar graphs of average tumor volume in each PARP inhibitor treatment group after switching to JPI-547 on day 60 (*n* = 5). **e** Images of harvested tumor tissues. **f** Immunoblot analysis of tumor tissues showing comparative expressions of tankyrase, MERIT40, and RAD51 in each group. **g** Immunohistochemistry staining of RAD51 in WJO-003-O2 engraft tumors of indicated groups. Staining images were collected at 400x magnification. The bar graph represented the RAD51 protein expression in 5 random, non-overlapped fields. Independent sample t-test: *p < 0.05, **p < 0.01, ***p < 0.001.

During the individual PARP inhibitors treatment, on day 21, JPI-547 inhibited tumor growth more than the other first-generation PARP inhibitors (Fig. 5a, b). As observed in PARP inhibitor-sensitive PDTX experiments, tumor growth was significantly suppressed in the groups that switched from first-generation PARP inhibitor treatments to the JPI-547 treatment, compared to those that continued receiving the same first-generation PARP inhibitor. (Fig. 5c, d). Moreover, no discernible weight loss was observed during the administration period, indicating no systemic drug toxicity (Supplementary Fig. 4A).

Mice were sacrificed and tumors were excised on day 60 of drug treatment and protein expression in the tumor tissue was measured (Fig. 5e, f and Supplementary Fig. 4B). Switching from first-generation PARP inhibitors to JPI-547 suppressed PAR, tankyrase, MERIT40, and RAD51 protein expression measured by immunoblot in both the PARP inhibitor-resistant in parallel with the in vitro findings. The increased suppression of RAD51 induced by JPI-547 compared with the control or other PARP inhibitor groups was confirmed by IHC (*p* < 0.001) (Fig. 5g). Furthermore, switch-to-JPI-547 groups showed significant RAD51 suppression compared with non-switch groups which received only first-generation PARP inhibitors. Collectively, the in vivo experiments demonstrated that JPI-547 showed greater antitumor activity than the other first-generation PARP inhibitors in both the olaparib-resistant and olaparib-sensitive models.

### High RAD51 is associated with poor prognosis in ovarian cancer

**Fig. 6:**
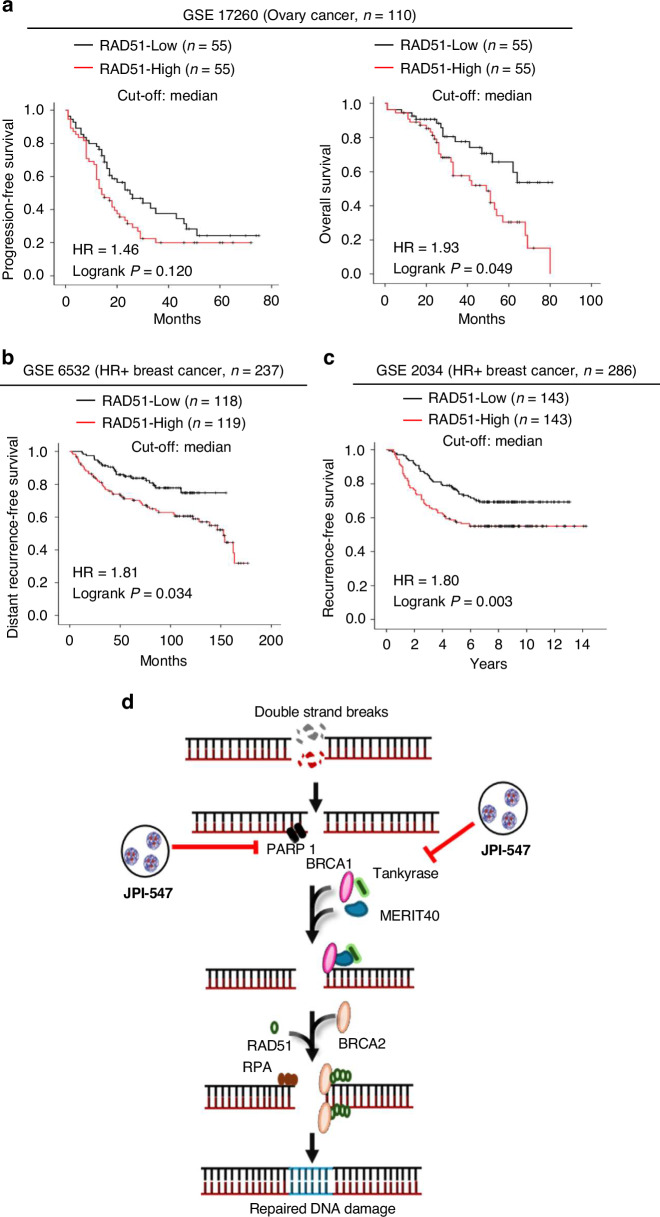
High RAD51 is associated with poor prognosis in ovarian cancer. **a** Kaplan-Meier survival curves of PFS and OS in ovarian cancer according to relative RAD51 mRNA expression from public mRNA microarray data set of GSE 17260 analysis was performed using self-downloaded processed data. **b**, **c** Kaplan-Meier survival curves of distant RFS and RFS in HR+ breast cancer according to relative RAD51 mRNA expression form public mRNA microarray data set of GSE 6532, and **c** GSE 2034 analysis was performed using self-downloaded processed data. **d** Schematic illustration showing the proposed mechanism of action of JPI-547 to overcome olaparib inhibitor resistance.

The impact of RAD51 expression on prognosis was determined using public mRNA expression datasets. The cut-offs were set at the median RAD51 expression levels for GSE 17260, GSE 6352, and GSE 2034. In the ovarian cancer dataset (GSE17260), the PFS and OS were shorter in the RAD51-High group (≥median) compared with survival in the RAD51-Low group (<median) (Fig. 6a). RAD51-High was a poor prognostic factor in the multivariate analysis adjusted by stage, tumor grade, and surgery type (Table S4). Likewise, in breast cancer data sets (GSE6532, GSE2034), RAD51-High’ patients also showed a poor prognosis in terms of distant RFS or RFS (Fig. 6b, c) which was confirmed in the multivariate analysis, adjusted by estrogen receptor status and stage (Table S5 and S6). Overall, the clinical relevance of RAD51 as a therapeutic target was supported by the significant association between RAD51 overexpression and poor prognosis in these public mRNA databases.

Our proposed mechanism of JPI-547 is shown in Fig. 6d. JPI-547 treatment suppressed the tankyrase-MERIT40-RAD51 axis in PARP inhibitor-sensitive and resistant cancer cells. In summary, JPI-547 treatment enhances the antitumor efficacy through targeting PARP1/2 and tankyrase.

## Discussion

PARP inhibitors are highly effective in treating BRCA1/2-mutated or HR-deficient cancers, including ovarian and breast cancers. However, acquired resistance to PARP inhibitors is a major obstacle to treatment, and a comprehensive understanding of the underlying mechanisms of PARP inhibitor resistance is needed to solve this problem. In this study, we established olaparib-resistant cell lines and PDTX models to evaluate the mechanisms of PARP inhibitor resistance. We demonstrated that PARP inhibitor resistance in BRCA1/2-mutated ovarian and breast cancers is associated with the upregulation of RAD51, which is central to DNA replication and damage repair in HR. We also demonstrated that JPI-547 engages both PARP1/2 and tankyrase targets and is associated with enhanced antitumor efficacy in preclinical models, accompanied by suppression of RAD51 within the HR pathway. Although the present study did not include tankyrase-specific genetic perturbation approaches, such as knockdown or rescue experiments, which would be required to definitively establish functional dependency on tankyrase inhibition, our biochemical and pharmacological data suggest potential engagement of both PARP1/2 and tankyrase by JPI-547. Future studies incorporating genetic validation will be important to further delineate the contribution of tankyrase inhibition to the observed antitumor effects.

Interestingly, our analysis also revealed an unexpected increase in sensitivity to niraparib in olaparib-resistant cell lines. Notably, in PEO1-OR cells, niraparib exhibited slightly greater cytotoxicity than JPI-547. This observation suggests that distinct mechanisms of resistance may differentially affect susceptibility to various PARP inhibitors. Previous work by Drost et al further demonstrated that the mechanisms of olaparib resistance in PEO1-derived high-grade serous ovarian cancer models are strongly influenced by BRCA2 functionality and treatment exposure paradigms, suggesting that BRCA2-specific restoration of HR repair can critically modulate PARP inhibitor response [35]. Our in vitro study demonstrated that niraparib exerted greater cytotoxicity in olaparib-resistant cells, suggesting that certain resistance mechanisms remain susceptible to niraparib-induced replication stress. Nevertheless, this in vitro enhanced sensitivity to niraparib was not recapitulated in olaparib-resistant PDTX models, implying that factors within the tumor microenvironment or alternative resistance mechanisms may mitigate niraparib’s efficacy in vivo. These findings highlight the multifaceted nature of PARP inhibitor resistance and emphasize the need for further mechanistic studies to delineate how specific modes of resistance influence the response to subsequent PARP inhibitors.

Given the widespread clinical use of PARP inhibitors across multiple cancer types, our olaparib-resistant preclinical models may serve as valuable tools for identifying molecular determinants of resistance and for developing agents capable of restoring drug sensitivity. As a recombinase essential for DNA replication and HR, RAD51 forms helical nucleoprotein filaments on single- or double-stranded DNA substrates [36, 37], facilitating DNA strand exchange and thereby promoting HR-mediated repair [37]. Thus, RAD51 induces PARP inhibitor resistance by either reinstating the HR pathway or stabilizing the replication forks [28, 38]. To overcome PARP inhibitor resistance, researchers have explored various mechanisms underlying PARP inhibitor resistance and predictive biomarkers for the resistance, as well. Previous studies suggested that RAD51 is a potential novel predictive biomarker for drug resistance to platinum-based drugs and PARP inhibitors [39–41]. In addition to that, there was evidence that RAD51 would have a mechanistically causal relationship with PARP inhibitor resistance rather than be a bystander biomarker [42–44]. Thus, investigating additional partners within the HR pathway that modulate RAD51 activity holds promise as a therapeutic strategy. To date, several small molecules targeting RAD51 have been reported. For example, B02 disrupts RAD51 binding to DNA, greatly increasing the sensitivity of cancer cells to genotoxic drugs [45]. Another RAD51 inhibitor, IBR2, blocks RAD51 multimerization and accelerates proteasome-mediated RAD51 protein degradation to overcome imatinib resistance in a chronic myeloid leukemia model [46]. The combined inhibition of RAD51 and PARP caused synthetic lethality in preclinical cell models of triple-negative breast cancer [47, 48]. In this study, JPI-547 inhibited both PARP1/2 and tankyrase pathways, which converged on RAD51 suppression. Taken together, our results and previous studies support RAD51 could be a therapeutic target to overcome PARP inhibitor resistance, as well as serve as a predictive biomarker of PARP inhibitor resistance.

In addition to targeting DNA repair–related factors such as RAD51, recent research has expanded to epigenetic mechanisms that regulate PARP inhibitor response. To address PARP inhibitor resistance, targeting epigenetic regulators of histone modifications, including both deacetylation and methylation, has emerged as a promising strategy. Recent studies have demonstrated that methylation of the BRCA1 promoter can induce sensitivity to PARP inhibitors [49]. Furthermore, the use of epigenetic modulators, such as EZH2 and HDAC inhibitors, has been shown to enhance PARP inhibitor sensitivity or overcome resistance by suppressing HR-related genes [50, 51]. Given these findings, further studies are warranted to investigate whether JPI-547 may also intersect with epigenetic regulatory pathways that influence DNA repair processes.

Although numerous preclinical studies have focused on RAD51 inhibitors, the mechanisms governing the regulation of RAD51 in the HR pathway were unclear. We identified a pathway involved in JPI-547-mediated modulation of RAD51 expression: the tankyrase-MERIT40-RAD51. Firstly, we demonstrated that tankyrase inhibition with the specific tankyrase inhibitor, XAV-939, suppressed the expression of MERIT40 in both olaparib-sensitive and resistant cells. We also verified the formation of the tankyrase-MERIT40 complex in ovarian PDTX models. In previous studies, tankyrase-MERIT40 complexes were recruited to double-stranded breaks (DSB), and then tankyrase recruited additional HR-associated proteins, including RAD51 and CtiP [16, 18]. Tankyrase was also reported to PARylate MERIT40. Recent studies showed that the accumulation of BRCA1 and RAD51 decreased by MERIT40 knockdown [52, 53]. Consistent with several reports, our results demonstrate that tankyrase inhibition downregulated MERIT40 and subsequent RAD51. Furthermore, JPI-547 remarkably decreased HR activity by inhibiting RAD51 through the tankyrase-MERIT40-RAD51 pathways.

Currently, no drugs to overcome PARP inhibitor resistance are clinically available. In a phase I trial, the PARP/tankyrase inhibitor E7449 (also known as 2X-121) exhibited antitumor activity; the objective response rate (ORR) in all patients was 4.9% (2 of 41 patients) and the ORR in BRCA1/2-mutated patients, none of whom received any PARP inhibitors, was 16.6% (1 of 6 patients). However, five of the 41 patients experienced dose-limiting toxicities (DLTs), including fatigue in 4, anaphylaxis in 1 [54, 55]. A phase II trial with E7449 in advanced ovarian cancer patients is underway (NTC03878849). In the phase I trial with JPI-547 briefly mentioned in the introduction section [19], 2 of 22 patients in the dose escalation phase experienced DLTs (elevated alanine aminotransferase or aspartate transferase), and the most common treatment-related adverse event included anemia, thrombocytopenia, and neutropenia. All adverse events were manageable. In the dose expansion phase, JPI-547 showed a promising ORR of 28.2% (11/39) in patients with BRCA/HRD-positive solid tumors. In particular, one partial response was achieved in 5 olaparib-failing patients. Therefore, JPI-547 is being evaluated in ovarian cancer patients previously treated with a PARP inhibitor in a phase II trial (NCT05475184). Turning to another target, a phase I dose-escalation study with the RAD51 inhibitor, CYT-0851, was conducted [56]. No DLTs were observed in 22 patients. In a phase I/II trial, with advanced solid and hematologic cancer patients, CYT-0851 treatment resulted in an ORR of 20% (2 of 10 evaluable patients) (NCT03997968). A phase II trial of CYT-0851 in combination with gemcitabine, capecitabine, or rituximab/bendamustine is underway (NCT03997968). However, none of these drugs has demonstrated clinical efficacy in overcoming PARP inhibitor resistance yet. Based on the mechanism of action for JPI-547, which converges on RAD51 suppression and the preliminary clinical efficacy in olaparib-resistant patients in a phase I trial, JPI-547 is worthy of further clinical development to enhance the antitumor efficacy of PAPR inhibitors.

JPI-547 engages both PARP1/2 and tankyrase targets and effectively inhibits PARylation, accompanied by suppression of RAD51 and impairment of HR-associated processes. JPI-547 exhibited prominent antitumor activity in BRCA1/2-mutated olaparib-resistant ovarian and breast cancer preclinical models as well as olaparib-sensitive. Based on its synergistic mechanism targeting both PARP1/2 and tankyrase, as well as its clinical potential observed in a phase I trial, JPI-547 is worthy of further clinical development to enhance the efficacy of PAPR inhibitors.

## Supplementary information

Supplementary information

Western raw data

## Data Availability

All the data supporting the findings of this study are available within the paper and its Supplementary Information files. All other data supporting the findings of this study are available from the corresponding author upon reasonable request.
