# Supplementary material for: JPI-547, a novel dual inhibitor of PARP1/2 and tankyrase is more effective than first-generation PARP inhibitors in preclinical BRCA1/2-mutated cancer models
Source: Br J Cancer. 2026 May 6;135(4):503–17. doi: 10.1038/s41416-026-03411-3 (PMC13427753; doi:10.1038/s41416-026-03411-3)
Supplement: Supplementary file 1 — Supplementary information [file 41416_2026_3411_MOESM1_ESM.docx]

**Supporting Information for**

**JPI-547, a novel dual inhibitor of PARP1/2 and tankyrase is more effective than first-generation PARP inhibitors in preclinical BRCA1/2-mutated cancer models**

Min Sil Kang^1^, Nar Bahadur Katuwal^1^, Mithun Ghosh^1^, Sa-Deok Hong^1^, Yeong Gyu Jeong^1^, Seong Min Park^1^, Tae Hoen Kim^3^, Seul-Gi Kim^2^, Seung Ryeol Lee^4^, Yong Wha Moon^2*^

^1^Department of Biomedical Science, The Graduate School, CHA University, Seongnam-si, Republic of Korea

^2^Hematology and Oncology, Department of Internal Medicine, CHA Bundang Medical Center, CHA University, Seongnam-si, Republic of Korea

^3^Department of Pathology, CHA Bundang Medical Center, CHA University, Seongnam-si, Republic of Korea

^4^Department of Urology, CHA Bundang Medical Center, CHA University, Seongnam-si, Korea

Corresponding author: Yong Wha Moon

E-mail: [ymoon@cha.ac.kr](mailto:ymoon@cha.ac.kr)

**This file includes:** Supplementary Figures 1 to 4, Tables S1 to S6.

**Supplementary Figures**

**
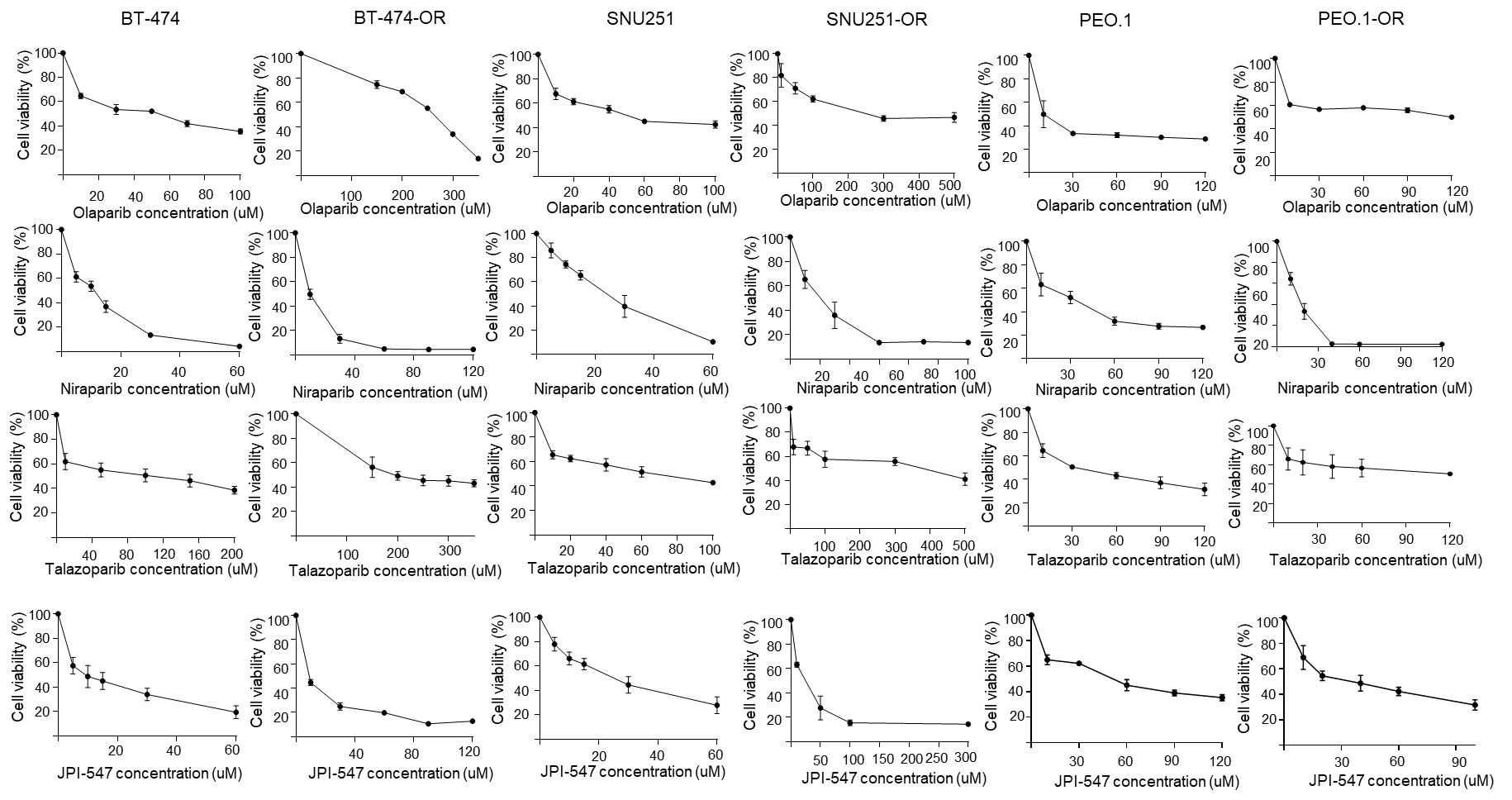
**

**Supplemental Figure 1. Cell viability assay of parental and olaparib-resistant cells with various PARP inhibitors for 72 hours**

(A) Cell viability assay (MTT) of parental cells (BT-474, SNU251 and PEO.1) and their respective olaparib-resistant cells (BT-474-OR, SNU251-OR and PEO.1) after 72 hours of treatment with the indicated PARP inhibitors.


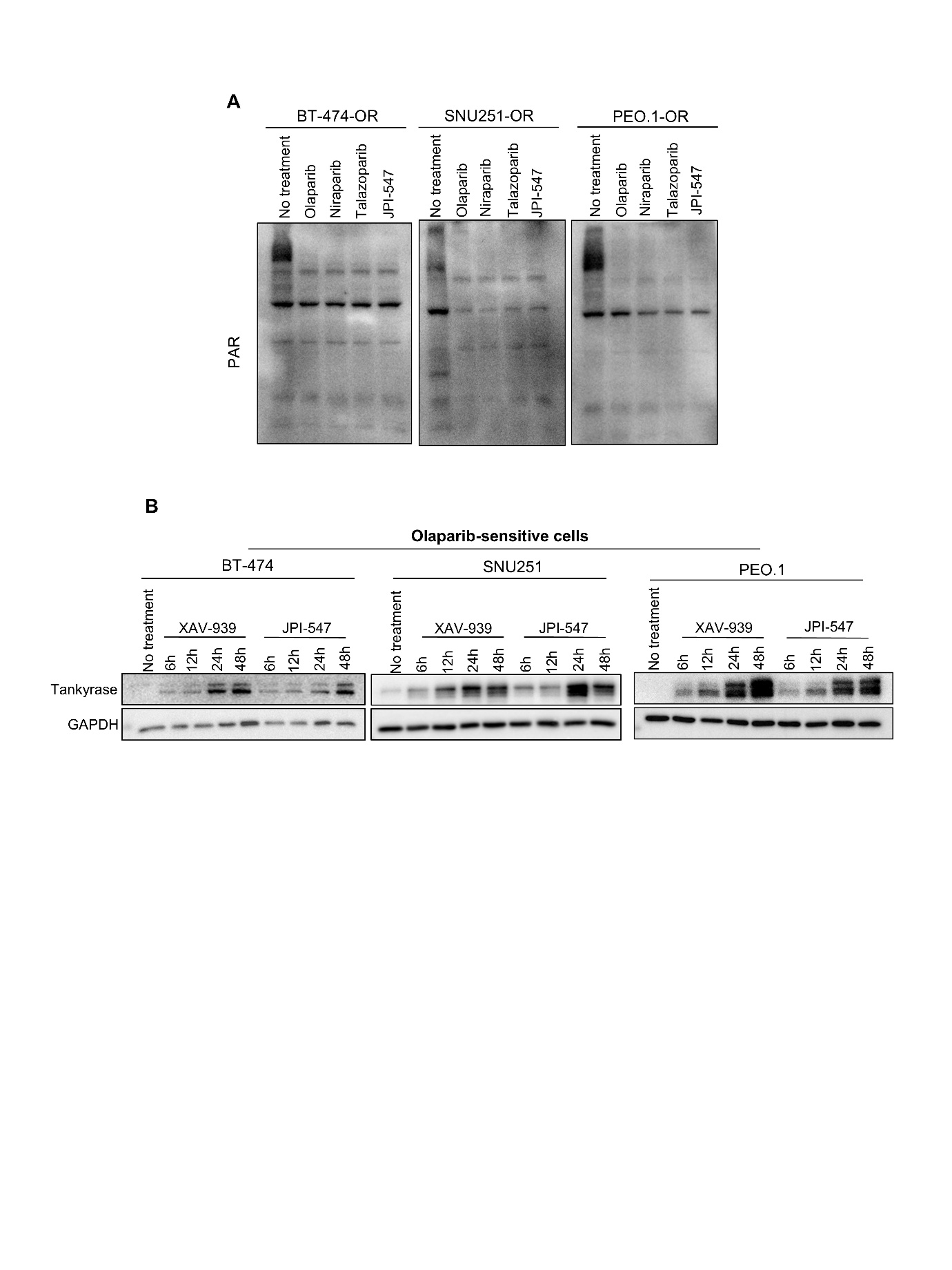


**Supplemental Figure 2. Comparing tankyrase auto-PARylation inhibition in various**

**BRCA1/2-mutated cancer cells.**

(A) Representative immunoblot analyses showing the detection of PAR (poly ADP-ribose) in olaparib-resistant cells (BT-474-OR, SNU251-OR, PEO.1-OR) after treatment with various PARP inhibitors at the JPI-547 IC_50_ concentration specific to each olaparib-resistant cell for 0.5h.

(B) Comparative analysis of tankyrase auto-PARylation inhibition activity between JPI-547 and XAV-939 in BRCA1/2-mutated cancer cells (BT-474, SNU251, and PEO.1).


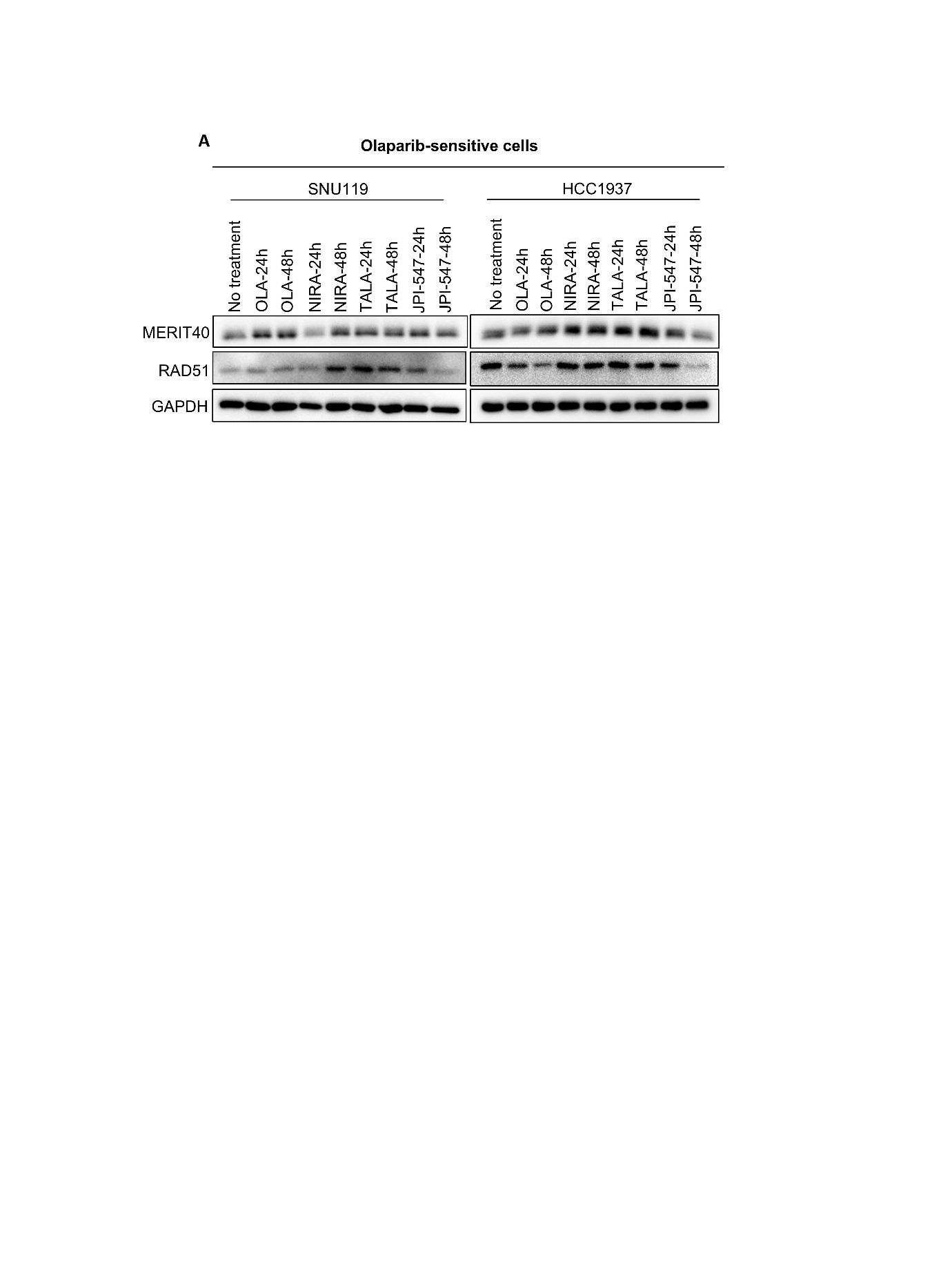


**Supplemental Figure 3. JPI-547 Inhibits HR-associated protein expressions.**

(A) Immunoblot analysis of MERIT40, and RAD51 expression in other BRCA1/2-mutated cancer cells (SNU119 and HCC1937) after treatment with the indicated PARP inhibitors for 24 and 48 hours at respective IC_50_ concentration (which were measured at 72 hours, results not shown).

**
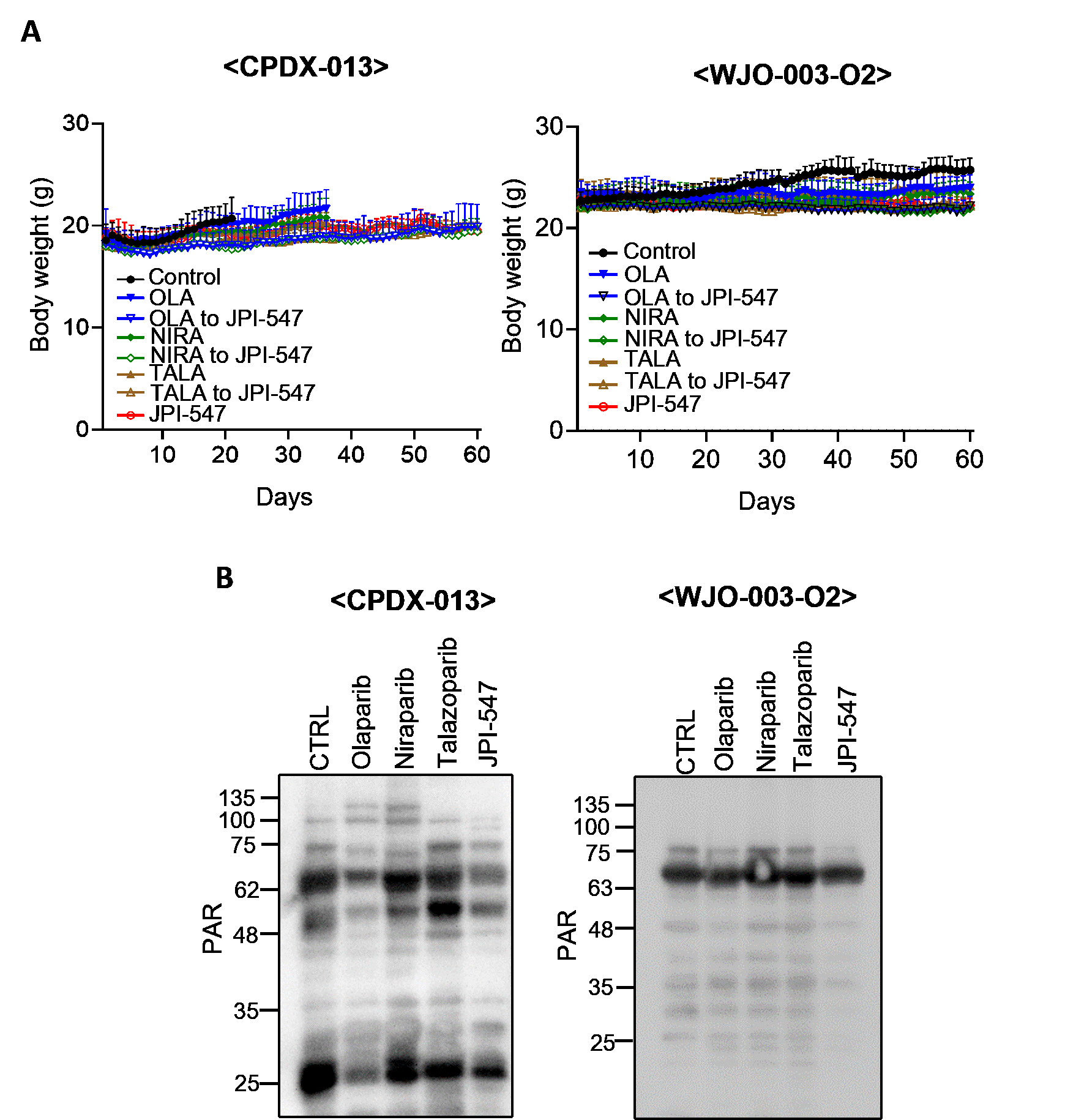
**

**Supplemental Figure 4.** (A) Average body weight of mice in two ovarian PDTX models; CDPX-013 and WJO-003-O2. Error bars represent the SD of 5 mice per group.

(B) Immunoblot analysis of PAR in two ovarian PDTX models; CDPX-013 and WJO-003-O2.

**Supplementary Tables**

| Supplemental Table 1. List of primers used for qRT-PCR | | | |
| --- | --- | --- | --- |
| Primer name | Orientation | Sequence | |
| RAD51 | Sense | GAGAAGGTGTGGTGGTGTTATG | |
|  | Anti-sense | ACTCACAGGGCAGTCTCTATAC | |
| Beta-actin | Sense | AGAGCTACGAGCTGCCTGAC | |
|  | Anti-sense | AGCACTGTGTTGGCGTACAG | |
|  |  | |  |

| Supplemental Table 2. Primary and secondary antibodies | | | | |
| --- | --- | --- | --- | --- |
| Antibody | Host species | Dilution | Catalog no, Company | Application |
| RAD51 | Rabbit | 1:800 | ab133534, abcam | IHC |
| RAD51 | Rabbit | 1:1000 | ab133534, abcam | WB |
| MERIT40 | Rabbit | 1:200 | A302-516A, BEATHYL | IP |
| MERIT40 | Rabbit | 1:1000,  1:200 | 12711, Cell Signaling | WB, IP |
| Tankyrase | Mouse | 1:1000 | sc-36589, Santa Cruz | WB |
| PARP | Rabbit | 1:1000 | 9532, Cell Signaling | WB |
| BRCA1 | Rabbit | 1:1000 | 14823, Cell Signaling | WB |
| AXIN1 | Rabbit | 1:1000 | 2087, Cell Signaling | WB |
| Active B-catenin | Rabbit | 1:1000 | 8814, Cell Signaling | WB |
| Cyclin D1 | Rabbit | 1:1000 | 55506, Cell Signaling | WB |
| Histone H3 | Rabbit | 1:1000 | ab1791 | WB |
| GAPDH | Rabbit | 1:1000 | 2118, Cell Signaling | WB |
| Anti-Rabbit HRP | Goat | 1:5000 | GTX213110-01, GeneTex | WB |
| Anti-Mouse HRP | Goat | 1:5000 | GTX213111-01, GeneTex | WB |

| Supplemental Table 3. A list of 20 BRCA-mutated breast cancer cell lines in the GDSC database and olaparib IC_50_. | | | |
| --- | --- | --- | --- |
| Cell line | Cosmic ID | Cancer type | Olaparib IC_50_ (µM) |
| HCC1954 | 749709 | breast | 4.5 |
| HCC1395 | 749712 | breast | 3.80 |
| HCC1937 | 749714 | breast | 3.96 |
| HCC2157 | 749715 | breast | 4.72 |
| HCC38 | 749717 | breast | 4.28 |
| BT-549 | 905951 | breast | 3.49 |
| BT-20 | 906801 | breast | 4.55 |
| HCC1569 | 907046 | breast | 3.28 |
| MDA-MB-3 | 9908121 | breast | 4.17 |
| MDA-MB-4 | 9908123 | breast | 2.36 |
| MRK-nu-1 | 908151 | breast | 4.12 |
| UACC-893 | 909778 | breast | 2.57 |
| ZR-75-30 | 909907 | breast | 6.21 |
| CAL-85-1 | 910852 | breast | 3.24 |
| CAL-51 | 910927 | breast | 1.35 |
| BT-474 | 946359 | breast | 3.85 |
| BT-483 | 949093 | breast | 6.73 |
| MDA-MB-4 | 1240172 | breast | 1.88 |
| HCC1428 | 1290905 | breast | 4.35 |
| JIMT-1 | 1298157 | breast | 3.24 |

| Supplemental Table 4. Univariate and multivariate analysis of prognosticators for PFS and OS in GSE 17260 | | | | | | | | | | | |
| --- | --- | --- | --- | --- | --- | --- | --- | --- | --- | --- | --- |
|  | |  | PFS | | | | | OS | | | |
| N=110 | | Total patients | Univariate | | Multivariate | | | Univariate | | Multivariate | |
| Factor | | N (%) | Median  PFS (mo) | *p*-  value | HR  (95% CI) | | *p*-value | Median  OS  (mo) | *p*-value | HR (95% CI) | *p*-value |
| Stage |  |  |  | 0.003 |  | | 0.201 |  | 0.415 |  | 0.921 |
|  | IIIA-IIIB | 24 (21.8%) | 33.5 |  | 1 | |  | 63 |  | 1 |  |
|  | IIIC | 69 (62.7%) | 17 |  | 1.27 (0.61~  2.64) | | 0.513 | 50 |  | 1.07 (0.45~2.52) | 0.886 |
|  | IV | 17 (15.5%) | 13.5 |  | 1.98 (0.85~  4.60) | | 0.112 | 44 |  | 1.22 (0.43~3.47) | 0.712 |
| Tumor  grade |  |  |  | 0.077 |  | | 0.822 |  | 0.045 |  | 0.333 |
|  | 1 | 26 (23.6%) | 27.5 |  | 1 | |  | 65 |  | 1 |  |
|  | II | 41 (37.3%) | 18 |  | 1.11 (0.57~  2.13) | 0.766 | | 59.5 |  | 1.40 (0.55~3.54) | 0.478 |
|  | III | 43 (39.1%) | 14.5 |  | 1.23 (0.63~  2.40) | | 0.546 | 46 |  | 1.93 (0.77~4.88) | 0.163 |
| Surgery |  |  |  | <0.001 |  | | 0.001 |  | 0.013 |  | 0.220 |
|  | Optimal debulking | 57 (51.8%) | 30 |  | 1 | |  | 67.5 |  | 1 |  |
|  | Suboptimal debulking | 53 (48.2%) | 14 |  | 2.46 (1.44~  4.22) | | 0.001 | 46 |  | 1.55  (0.77-3.10) | 0.220 |
| RAD51 |  |  |  | 0.061 |  | | 0.394 |  | 0.006 |  | 0.049 |
|  | Low  (≤median) | 55 (50%) | 24 |  | 1 | |  | NR |  | 1 |  |
|  | High (>median) | 55 (50%) | 14 |  | 0.81 (0.5~  1.31) | | 0.394 | 45 |  | 1.93 (1.00~  3.74) | 0.049 |

Abbreviations: PFS (Progression-Free Survival), OS (Overall survival), HR (Hazard ratio), ER (Estrogen receptor), - (not provided), NR (Not Reached)

| Supplemental Table 5. Univariate and multivariate analysis of prognosticators for Distant RFS in GSE 6532 | | | | | | | |
| --- | --- | --- | --- | --- | --- | --- | --- |
| GSE6532 | | | Distant RFS | | | | |
| N=237 | | Total | Univariate | | | Multivariate | |
| Variables | | N (%) | 5Y-distant RFS rate | *p*-value | HR  (95% CI) | | *p*-value |
| ER status | Positive | 192 | 81.0% | 0.007 | 1 | |  |
|  | Negative | 39 | 65.1% |  | 1.76  (0.98-3.14) | | 0.057 |
|  | Unknown | 6 | - |  |  | |  |
| T stage | T1 | 112 | 84.2% | 0.051 | 1 | |  |
|  | T2-3 | 122 | 71.9% |  | 1.34  (0.83-2.17) | | 0.232 |
|  | Unknown | 3 | - |  |  | |  |
| N stage | N0 | 181 | 81.0% | 0.036 | 1 | |  |
|  | N1-3 | 51 | 65.5% |  | 2.02  (1.14-3.58) | | 0.016 |
|  | Unknown | 5 | - |  |  | |  |
| RAD51 | Low  (≤median) | 118 | 84.9% | 0.006 | 1 | |  |
|  | High (>median) | 119 | 71.2% |  | 1.81  (1.05-3.15) | | 0.034 |

Abbreviations: RFS (Recurrence- Free Survival), HR (Hazard ratio), ER (Estrogen receptor), - (not provided)

| Supplemental Table 6. Univariate and multivariate analysis of prognostic factors for RFS in GSE2034 | | | | | | | |
| --- | --- | --- | --- | --- | --- | --- | --- |
| GSE2034 | |  | RFS | | | | |
|  | | Total  N=237 | Total  N=286 | Univariate | | Multivariate | |
| Variables | | N (%) | N (%) | 5Y-RFS rate | *p*-value | HR  (95% CI) | *p*-value |
| ER status | Positive | 192 | 209 | 68.4% | 0.991 | 1 |  |
|  | Negative | 39 | 77 | 64.9% |  | 0.87 (0.55-1.35) | 0.543 |
|  | Unknown | 6 | 0 | - |  |  |  |
| RAD51 | Low (≤median) | 118 |  | 76.1% | 0.004 | 1 |  |
|  | High (>median) | 119 |  | 57.3% |  | 1.81  (1.22~2.68) | 0.003 |

Abbreviations: RFS (Recurrence- Free Survival), HR (Hazard ratio), ER (Estrogen receptor), - (not provided)
