# Supplementary figures and images for: JPI-547, a novel dual inhibitor of PARP1/2 and tankyrase is more effective than first-generation PARP inhibitors in preclinical BRCA1/2-mutated cancer models
Source: Br J Cancer. 2026 May 6;135(4):503–17. doi: 10.1038/s41416-026-03411-3 (PMC13427753; doi:10.1038/s41416-026-03411-3)

Fig. 1D

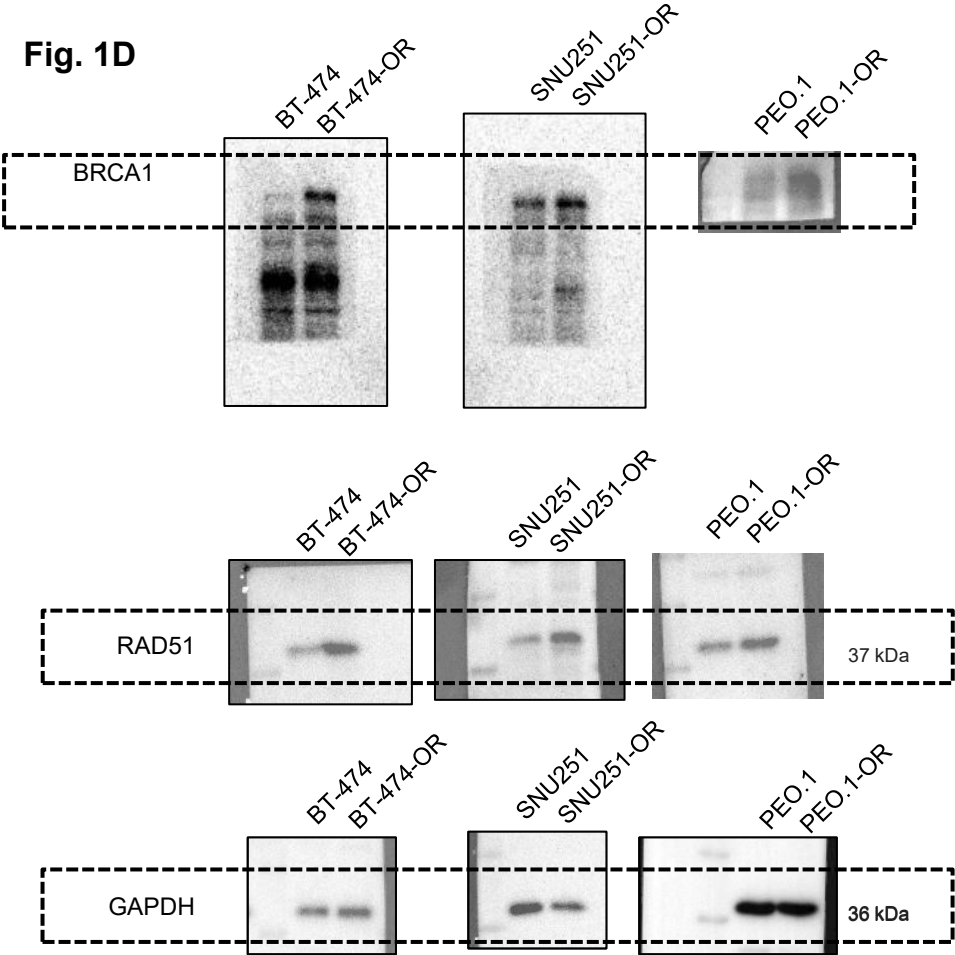

Fig. 1F

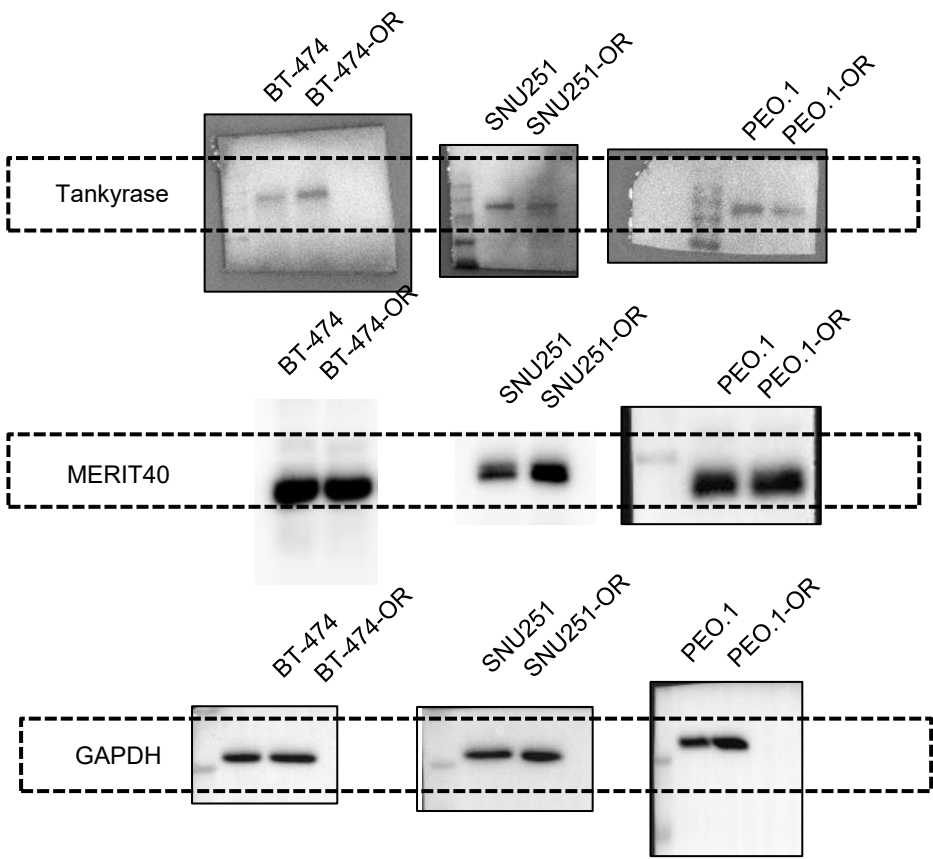

Fig. 1G

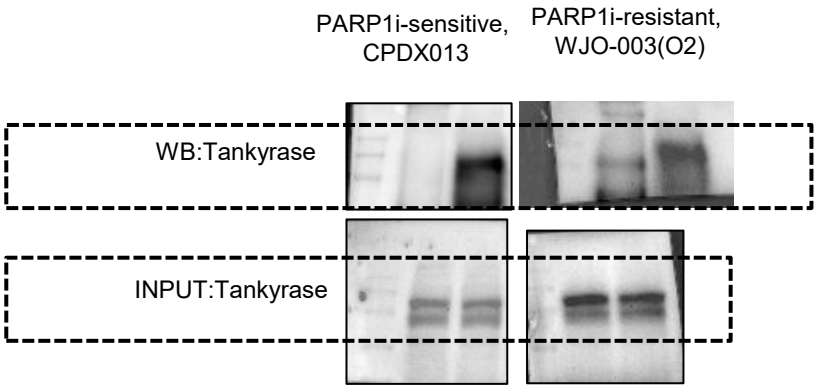

Fig. 2A

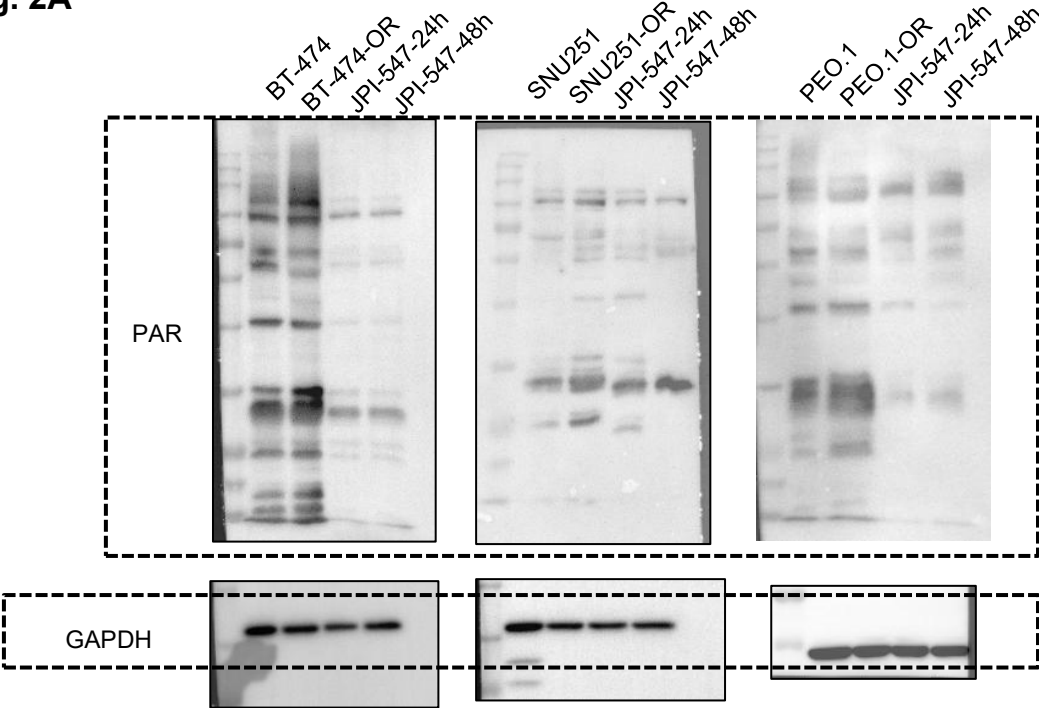

Fig. 2B

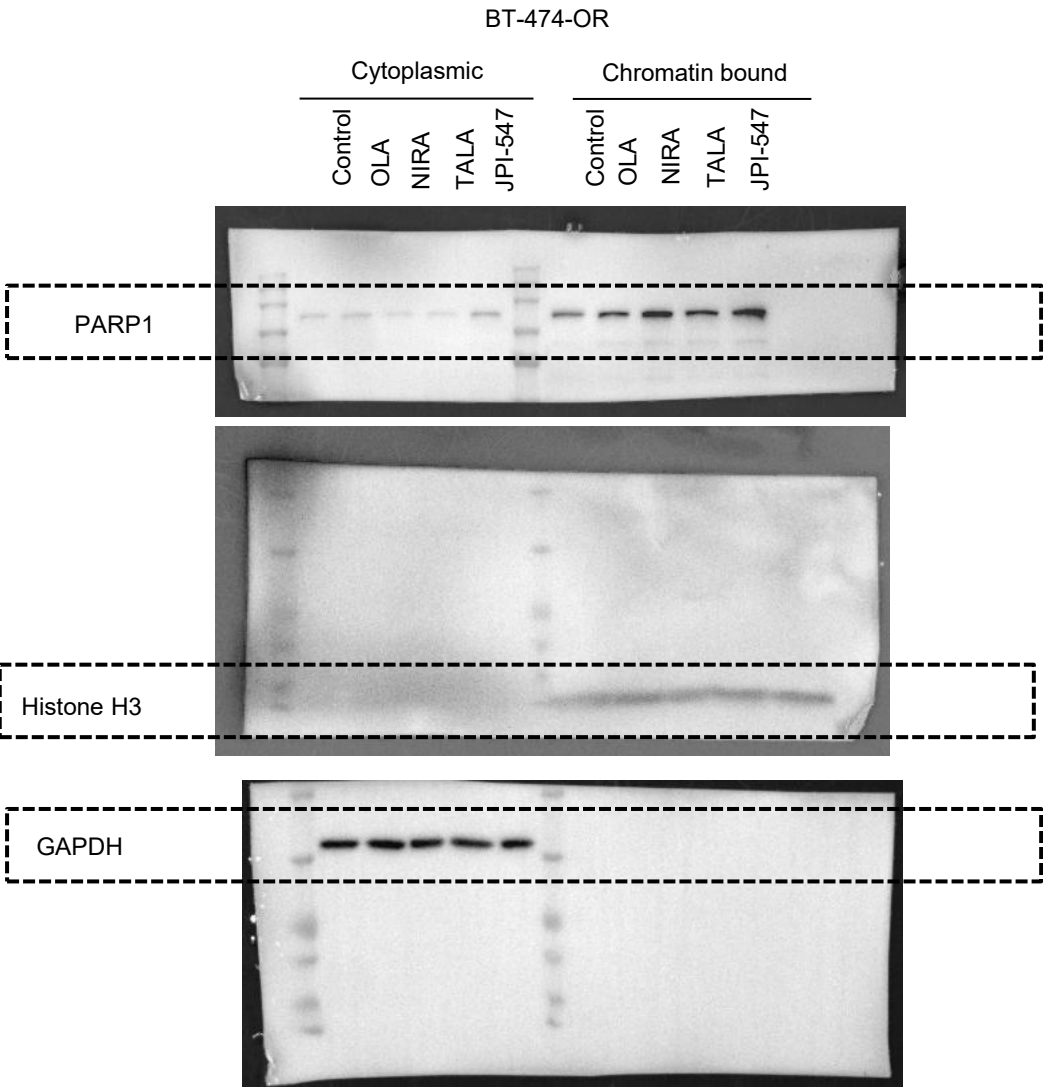

Fig. 2B

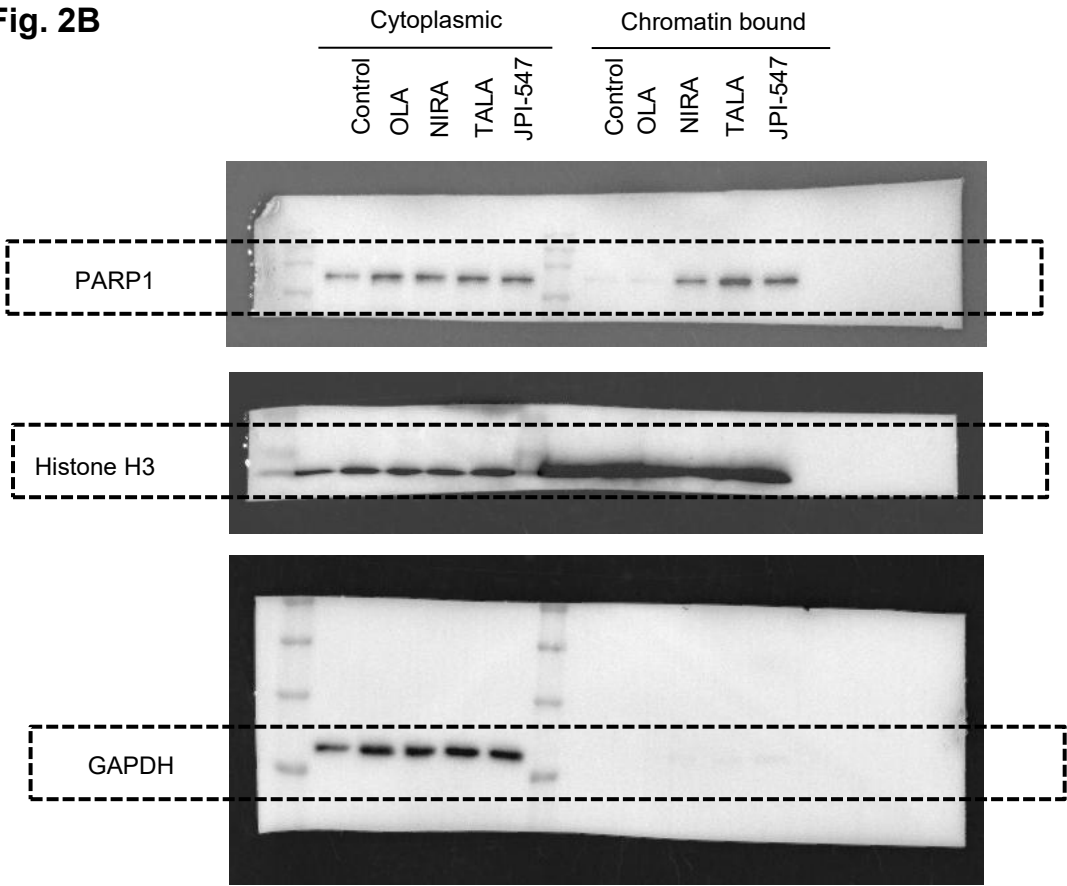

PEO.1-OR

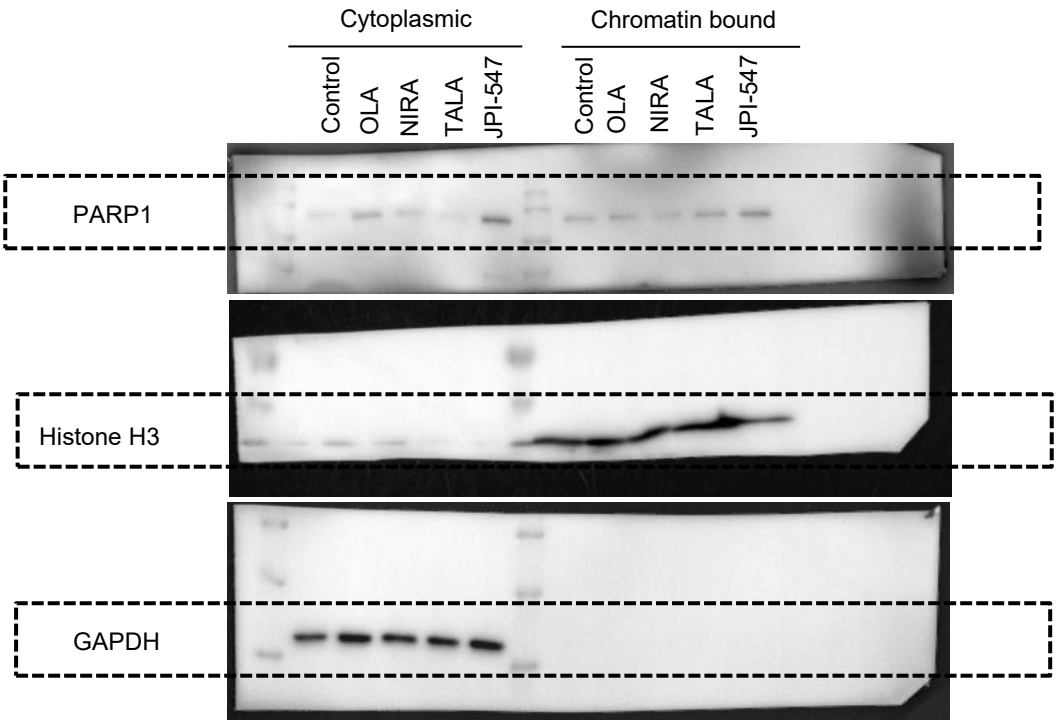

Fig. 2E

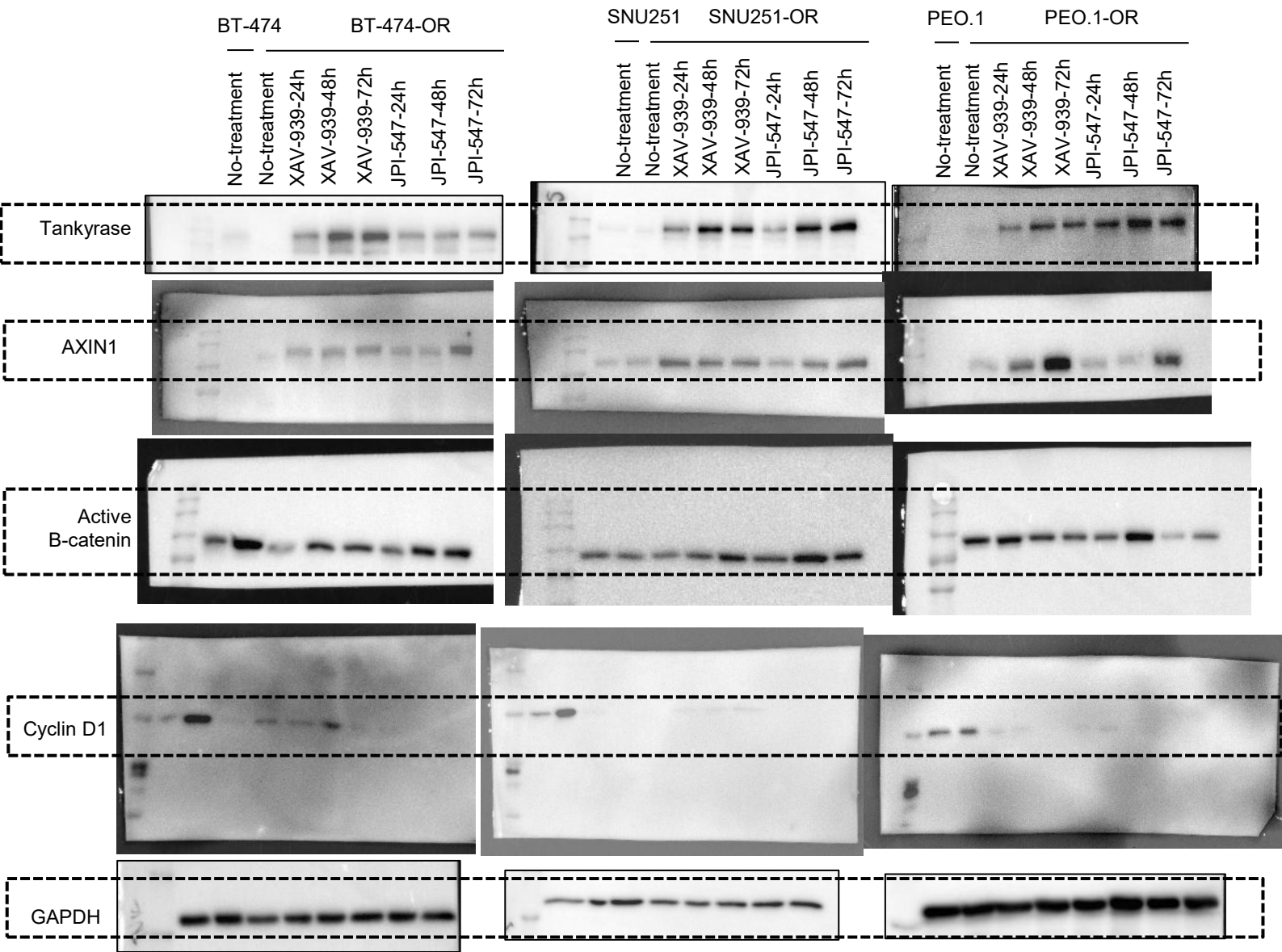

Fig. 2F

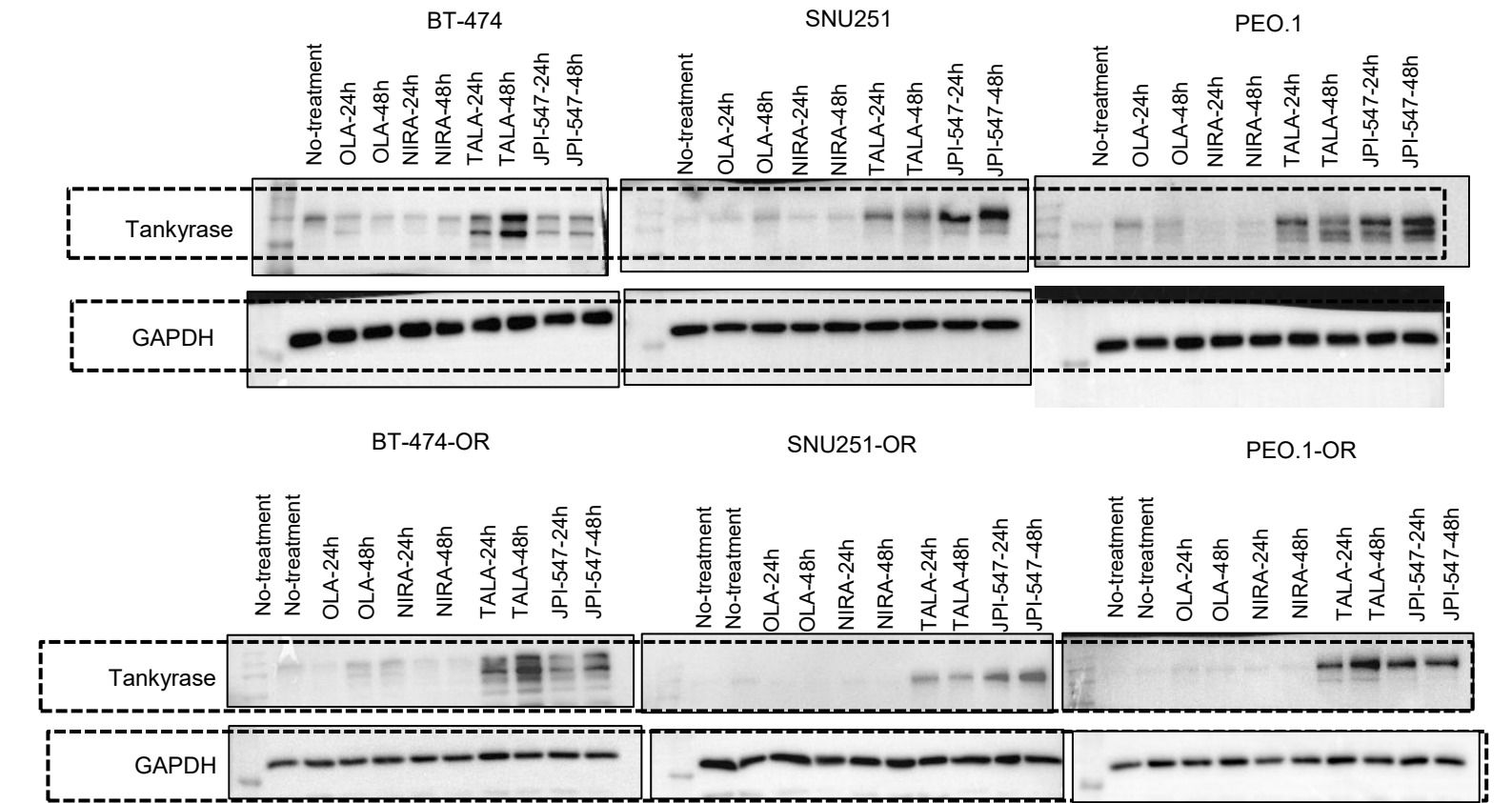

Fig. 3A

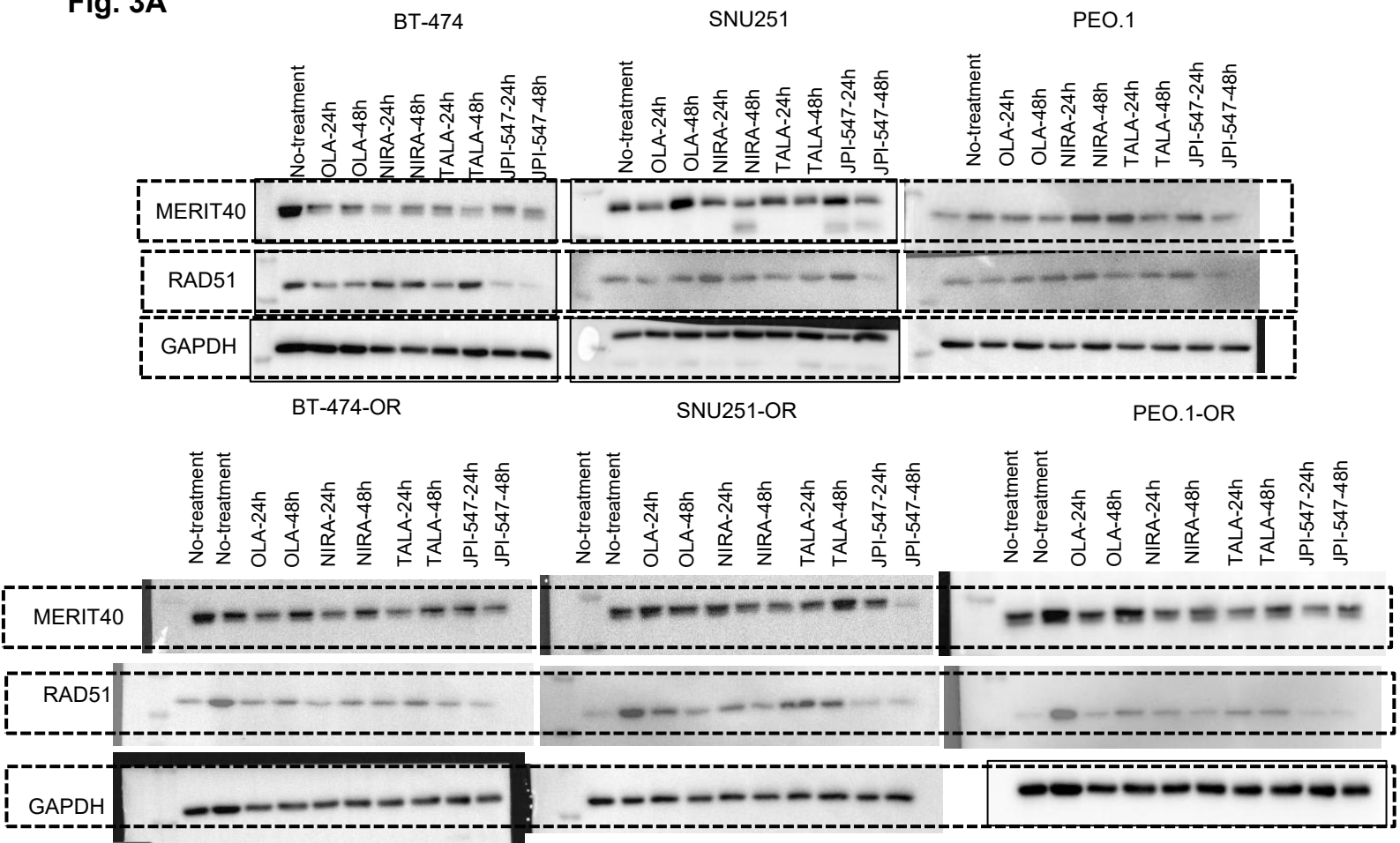

Fig. 3B

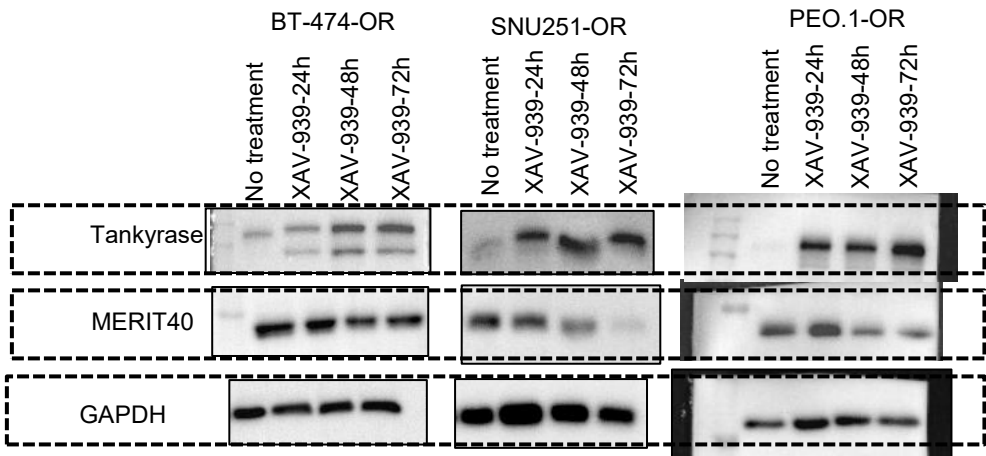

Fig. 3C

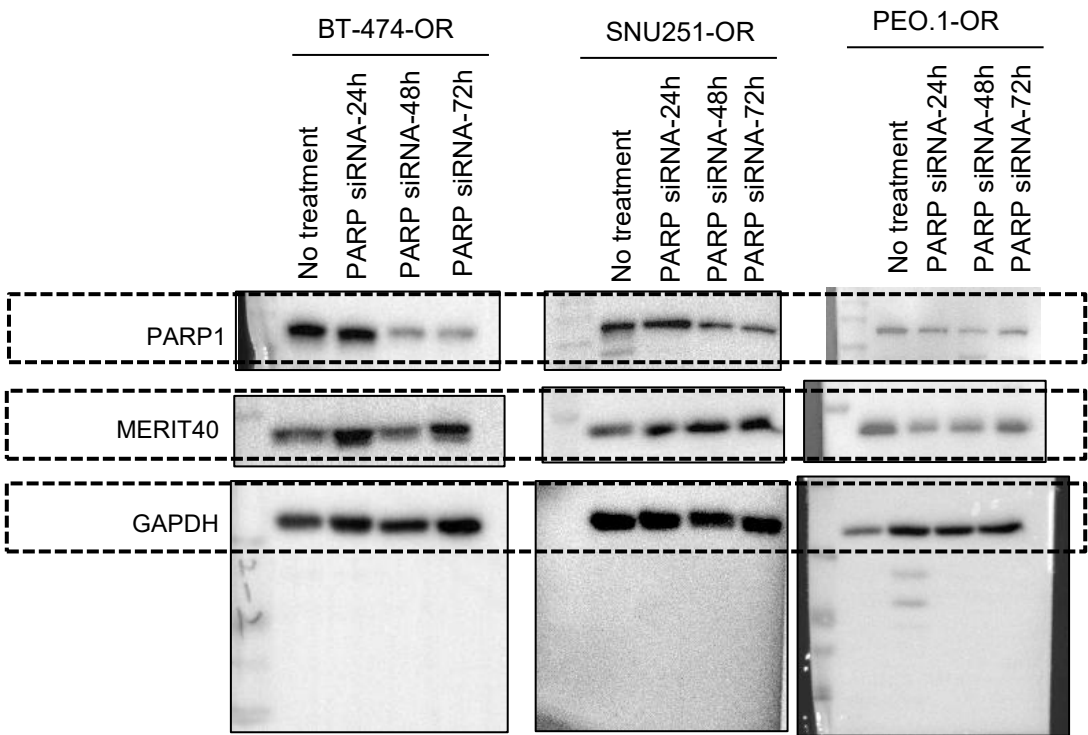



Suppl.Fig. S2A

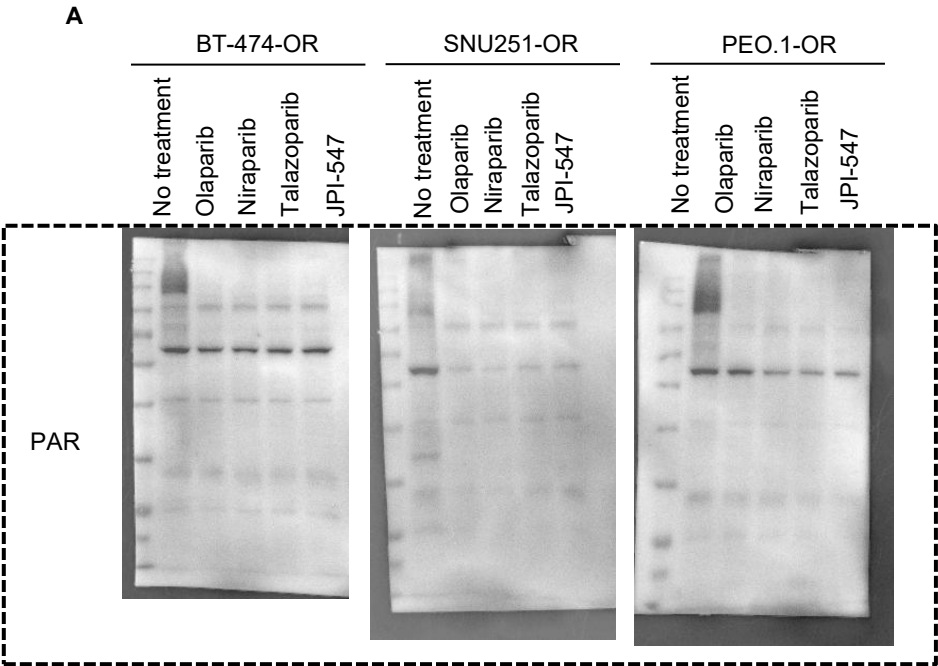

Suppl.Fig. S2B

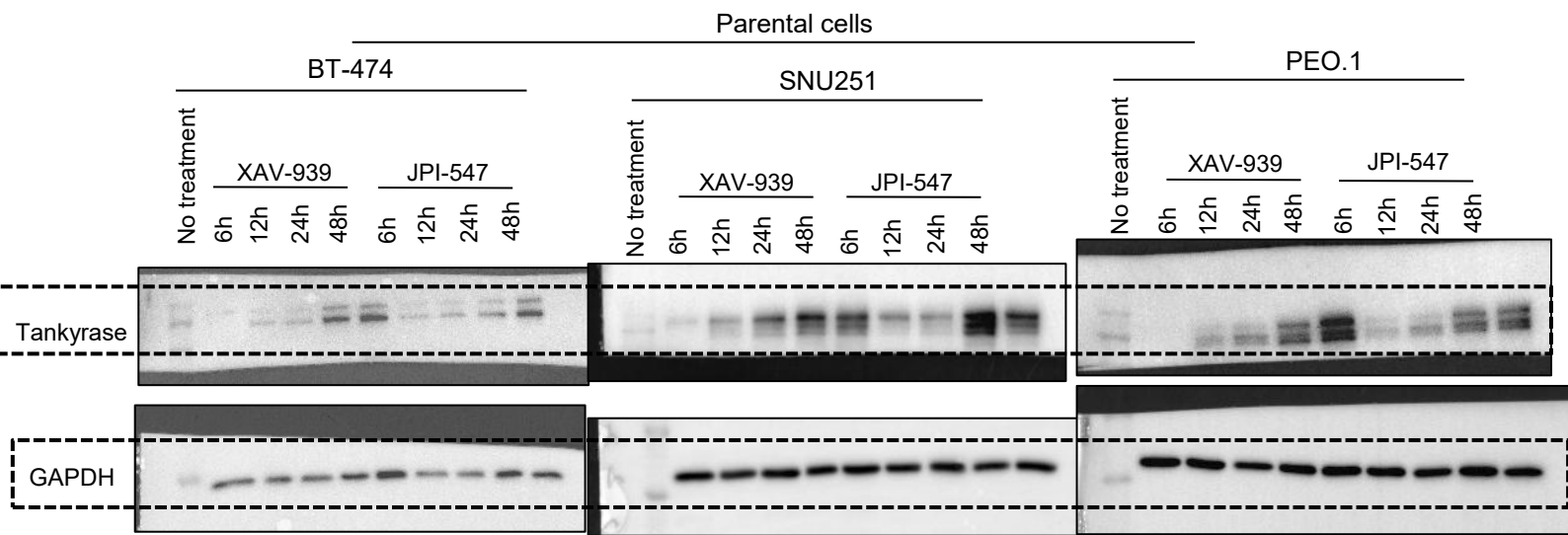

Suppl.Fig. S3A

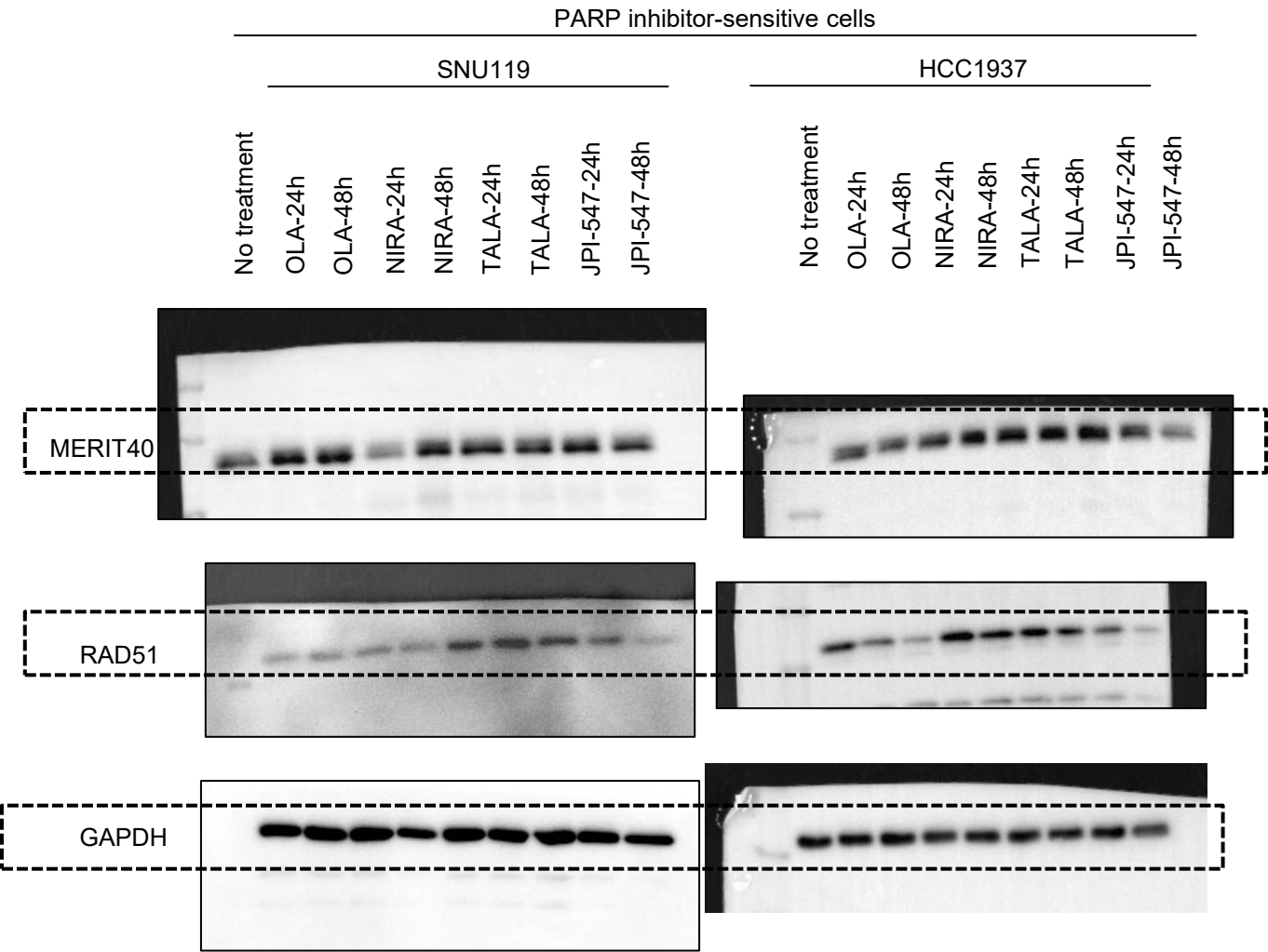

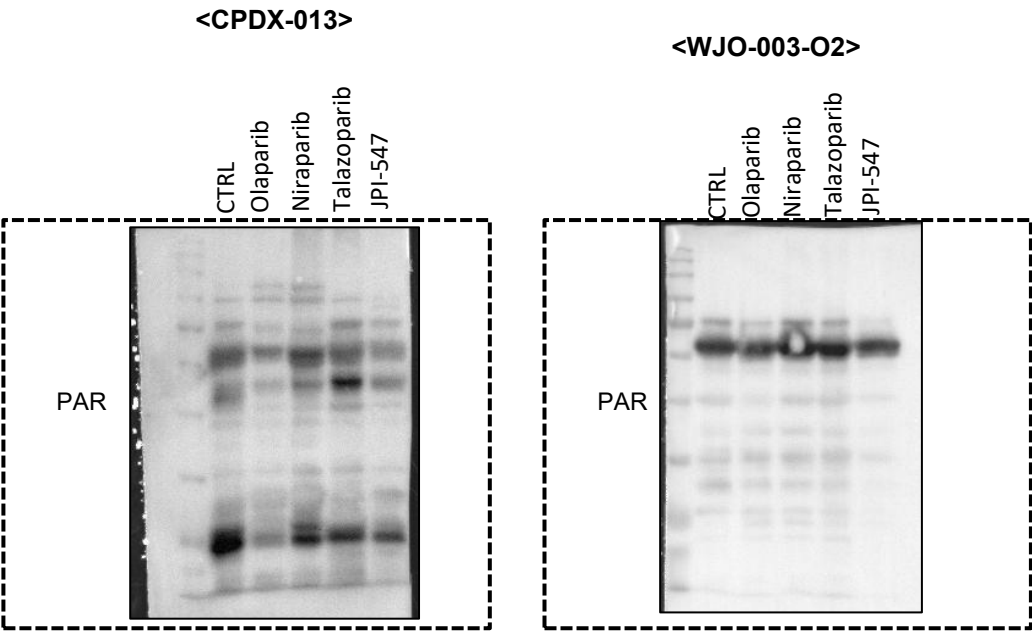

Supplement: Supplementary file 2 — Western raw data [file 41416_2026_3411_MOESM2_ESM.pdf]
